# Potent and selective inhibition of pathogenic viruses by engineered ubiquitin variants

**DOI:** 10.1371/journal.ppat.1006372

**Published:** 2017-05-18

**Authors:** Wei Zhang, Ben A. Bailey-Elkin, Robert C. M. Knaap, Baldeep Khare, Tim J. Dalebout, Garrett G. Johnson, Puck B. van Kasteren, Nigel J. McLeish, Jun Gu, Wenguang He, Marjolein Kikkert, Brian L. Mark, Sachdev S. Sidhu

**Affiliations:** 1 Donnelly Centre for Cellular and Biomolecular Research, Banting and Best Department of Medical Research, and Department of Molecular Genetics, University of Toronto, Toronto, Ontario, Canada; 2 Department of Microbiology, University of Manitoba, Winnipeg, Manitoba, Canada; 3 Department of Medical Microbiology, Leiden University Medical Center, Leiden, The Netherlands; Harvard Medical School, UNITED STATES

## Abstract

The recent Middle East respiratory syndrome coronavirus (MERS-CoV), Ebola and Zika virus outbreaks exemplify the continued threat of (re-)emerging viruses to human health, and our inability to rapidly develop effective therapeutic countermeasures. Many viruses, including MERS-CoV and the Crimean-Congo hemorrhagic fever virus (CCHFV) encode deubiquitinating (DUB) enzymes that are critical for viral replication and pathogenicity. They bind and remove ubiquitin (Ub) and interferon stimulated gene 15 (ISG15) from cellular proteins to suppress host antiviral innate immune responses. A variety of viral DUBs (vDUBs), including the MERS-CoV papain-like protease, are responsible for cleaving the viral replicase polyproteins during replication, and are thereby critical components of the viral replication cycle. Together, this makes vDUBs highly attractive antiviral drug targets. However, structural similarity between the catalytic cores of vDUBs and human DUBs complicates the development of selective small molecule vDUB inhibitors. We have thus developed an alternative strategy to target the vDUB activity through a rational protein design approach. Here, we report the use of phage-displayed ubiquitin variant (UbV) libraries to rapidly identify potent and highly selective protein-based inhibitors targeting the DUB domains of MERS-CoV and CCHFV. UbVs bound the vDUBs with high affinity and specificity to inhibit deubiquitination, deISGylation and in the case of MERS-CoV also viral replicative polyprotein processing. Co-crystallization studies further revealed critical molecular interactions between UbVs and MERS-CoV or CCHFV vDUBs, accounting for the observed binding specificity and high affinity. Finally, expression of UbVs during MERS-CoV infection reduced infectious progeny titers by more than four orders of magnitude, demonstrating the remarkable potency of UbVs as antiviral agents. Our results thereby establish a strategy to produce protein-based inhibitors that could protect against a diverse range of viruses by providing UbVs via mRNA or protein delivery technologies or through transgenic techniques.

## Author summary

Emerging viruses pose a tremendous challenge to human health. While vaccine-based approaches are desirable in terms of infection prevention in the longer term, alternative antiviral strategies are needed, especially when providing treatment options for infected patients during acute outbreaks. Here we applied protein engineering technology to target virus-encoded deubiquitinating enzymes of two viruses with significant impact on human health: Middle East respiratory syndrome coronavirus (MERS-CoV) and Crimean-Congo hemorrhagic fever virus (CCHFV). This resulted in the rapid identification of ubiquitin variant (UbV) inhibitors that bound with high affinity and specificity to the viral deubiquitinating enzymes. These proteins inhibited the catalytic activities of the deubiquitinating enzymes and almost completely blocked MERS-CoV infection. This work provides proof-of-principle that structurally diverse, virus-specific deubiquitinating enzymes can be selectively targeted through rational protein design technology, and therefore opens new avenues for quickly developed molecularly tailored therapy across a broad spectrum of viral pathogens that infect humans, livestock and plants.

## Introduction

Ubiquitination is a post-translational modification mediated by an enzyme cascade that results in the conjugation of ubiquitin (Ub) to cellular proteins [1, 2]. This process is regulated in part through activity of cellular deubiquitinating enzymes (DUBs), which remove Ub from cellular proteins [1, 2]. Given the essential role of the Ub system in regulating a large number of critical cellular processes, it is not surprising that viruses have acquired the means to modulate this system in order to promote infection and replication in host cells [3]. In particular, virus-encoded DUBs reverse the ubiquitination process to alter host signaling pathways critical to the induction of cellular antiviral and pro-inflammatory innate immune responses [3]. In addition to removing Ub molecules from host proteins, many viral DUBs (vDUBs) also remove the Ub-like protein interferon-stimulated gene 15 (ISG15) to further suppress antiviral responses [4, 5]. Importantly, a number of vDUBs also play an essential role in viral replication [4–6]. Together, the replicative and/or deubiquitinating activities of viral proteases contribute directly to pathogenesis during viral infection *in vivo* [7], making them ideal antiviral drug targets.

The Middle East respiratory syndrome coronavirus (MERS-CoV) and the severe acute respiratory syndrome coronavirus (SARS-CoV) viruses have caused significant concern globally due to their rapid emergence, high lethality rates in humans [8], and high potential for genetic recombination. Coronaviruses initially express their non-structural proteins (nsps) as large viral polyproteins, which are processed into functional domains by proteases encoded within the polyproteins to establish a viral replication-transcriptase complex. SARS- and MERS-CoV release nsp1-3 through the activity of a papain-like protease (PL^pro^) domain situated within nsp3, in a process that is indispensable for replication [4]. The chymotrypsin-like protease (3CL^pro^), corresponding to nsp5, is responsible for cleaving the remaining part of the polyproteins, releasing mature nsps [8]. Strikingly, coronaviral PL^pro^s also act as vDUBs to suppress host antiviral innate immune responses by targeting cellular Ub-conjugated substrates [9–14]. The CoV proteases are well-recognized drug targets, and since the emergence of these zoonotic CoVs research has focused on the identification and development of small molecule inhibitors targeting these enzymes [15, 16]. Another highly pathogenic virus that encodes a vDUB is the nairovirus Crimean-Congo hemorrhagic fever virus (CCHFV). The CCHFV vDUB domain is located within the large (L) segment of the genome, and has also been explicitly implicated in the evasion of host Ub- and ISG15-dependent innate immune responses [17].

Crystal structures of PL^pro^ from MERS- and SARS-CoV revealed that these proteases share structural similarity with cellular DUBs belonging to the ubiquitin-specific protease (USP) family [9, 18, 19]. Conversely, structural studies of nairovirus vDUBs revealed resemblance to the ovarian tumor (OTU) domain family of DUBs [20, 21]. In addition to these well characterized vDUBs, which are conserved in the coronavirus and nairovirus families respectively, numerous other vDUBs have been identified across diverse and distantly related virus lineages of significant concern to human health and agriculture. They include arteriviruses, herpesviruses, adenoviruses, picornaviruses, hepadnaviruses and tymoviruses, further emphasizing the broad potential utility of vDUB-specific antivirals [22].

The importance of vDUBs in viral replication and innate immune evasion make them attractive pharmacological targets, although their structural similarity with human DUBs has posed a significant challenge that complicates the successful development of highly selective small molecule vDUB inhibitors [23]. Despite intensive efforts, only a handful of inhibitors targeting vDUB proteases have been reported, and none have been approved for clinical use [24]. To meet this challenge, we have developed a strategy to target the vDUB activity as an antiviral approach by the rapid identification of virus-specific, protein-based vDUB inhibitors from a phage-displayed library of billions of Ub variants (UbVs). The exquisite specificity of identified UbVs has also enabled us to isolate UbVs that potently inhibit human Ub-binding proteins with equally high specificity [25, 26]. Here we describe UbVs that selectively block the deubiquitinating and deISGylating activities of MERS-CoV and CCHFV OTU vDUB domains. Importantly, UbVs specific for MERS-CoV abolished replicative polyprotein processing activities. Expression of MERS-CoV-specific UbVs during MERS-CoV infection reduced infectious progeny titers by more than four orders of magnitude, demonstrating the remarkable potency of UbVs as antiviral agents.

## Results

### Potent and selective UbV inhibitors of MERS-CoV and CCHFV vDUBs

**Fig 1:**
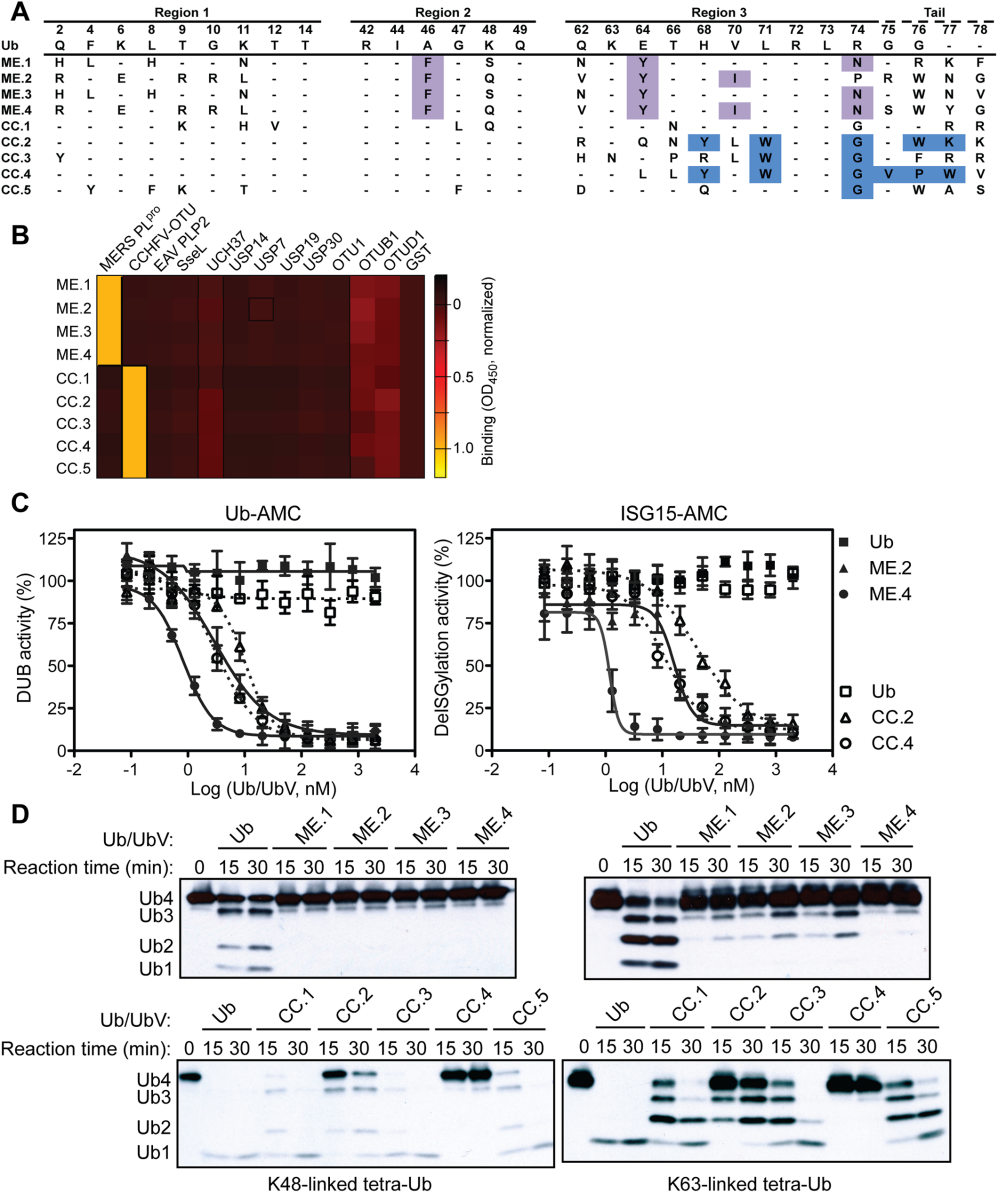
UbVs inhibit activity of MERS-CoV PL^pro^ and CCHFV OTU *in vitro*. (**A**) Sequences of UbVs that bind MERS-CoV or CCHFV vDUBs. Only regions subjected to diversification relative to Ub.wt in the phage-displayed library are shown. Amino acids discussed in the text are highlighted. (**B**) The binding specificities of phage-displayed UbVs *(y-axis*) are shown across a group of 12 DUBs *(x-axis)*, as assessed by phage ELISA. Sub-saturating concentrations of phage were added to immobilized proteins as indicated. Bound phages were detected by the addition of anti-M13-HRP and colorimetric development of TMB peroxidase substrate. The mean value of absorbance at 450 nm is shaded in a black-red-yellow gradient. (**C**) Inhibition of MERS-CoV PL^pro^ (solid lines) or CCHFV OTU (dashed lines) by the cognate UbVs shown as dose-response curves using Ub-AMC (*left*) or ISG15-AMC (*right*) as a substrate. The IC_50_ value was determined as the concentration of UbV that reduced proteolytic activity by 50% (**S1 Table**). The Ub.wt data obtained in the deISGylation assay cannot be fitted by GraphPad Prism so no lines are shown. (**D**) Effects of UbV inhibitors on vDUB activity against K48/K63 tetra-Ub substrates. Purified MERS-CoV PL^pro^ (*top panels*) or CCHFV OTU (*bottom panels*) was incubated with the indicated UbV or Ub.wt (negative control) and biotinylated tetra-Ub at 37°C for a time course of 30 minutes. Western blots were probed with ExtrAvidin-HRP (EA-HRP) to detect biotin-Ub. Inhibition of proteolysis was shown by a delay of appearance of the digestion products tri-Ub (Ub3), di-Ub (Ub2) and mono-Ub (Ub1).

The UbV library [25] was screened against the MERS-CoV PL^pro^ domain (MERS-CoV PL^pro^) and the CCHFV OTU domain (CCHFV OTU). Within three weeks, UbVs were identified that bound with high affinity to either MERS-CoV PL^pro^ (ME.1 to ME.4) or CCHFV OTU (CC.1 to CC.5) (**Fig 1A**). To confirm the specificity of the UbVs towards their cognate vDUBs, the phage-displayed UbVs were challenged against a diverse panel of 11 DUBs from several species representing distinct DUB families (USP, OTU, and ubiquitin C-terminal hydrolases (UCH)). All UbVs bound to their cognate viral proteins but not to any of the 11 additional DUBs (**Fig 1B**). To determine the binding affinity of each UbV, phage enzyme-linked immunosorbent assays (**S1 Fig**) and Bio-Layer Interferometry (BLI) measurements were performed (**S2 Table**). Each UbV was found to bind its cognate vDUB with affinities in the low to sub-nanomolar range, whereas wild-type Ub (Ub.wt) showed binding to MERS-CoV or CCHFV vDUBs only in the high micromolar range (**S1B Fig** and **S1 Table**). Consistent with the high affinities observed for the UbVs toward their respective vDUBs, each UbV also potently inhibited the deubiquitinating and deISGylating activities of MERS-CoV PL^pro^ or CCHFV OTU as measured using the fluorogenic substrates Ub-AMC or ISG15-AMC, respectively (**Fig 1C** and **S2 Fig**). The most potent inhibitors of MERS-CoV PL^pro^ and CCHFV OTU were ME.4 (deubiquitination IC_50_ = 0.8 nM and deISGlyation IC_50_ = 1.2 nM) and CC.4 (deubiquitination IC_50_ = 3.3 nM and deISGlyation IC_50_ = 11 nM), respectively (**S1 Table**). Furthermore, the UbVs were confirmed to inhibit processing of K48- and K63-linked tetra-Ub substrates by their respective vDUBs (**Fig 1D**).

### Structural basis for vDUB inhibition by UbVs

**Fig 2:**
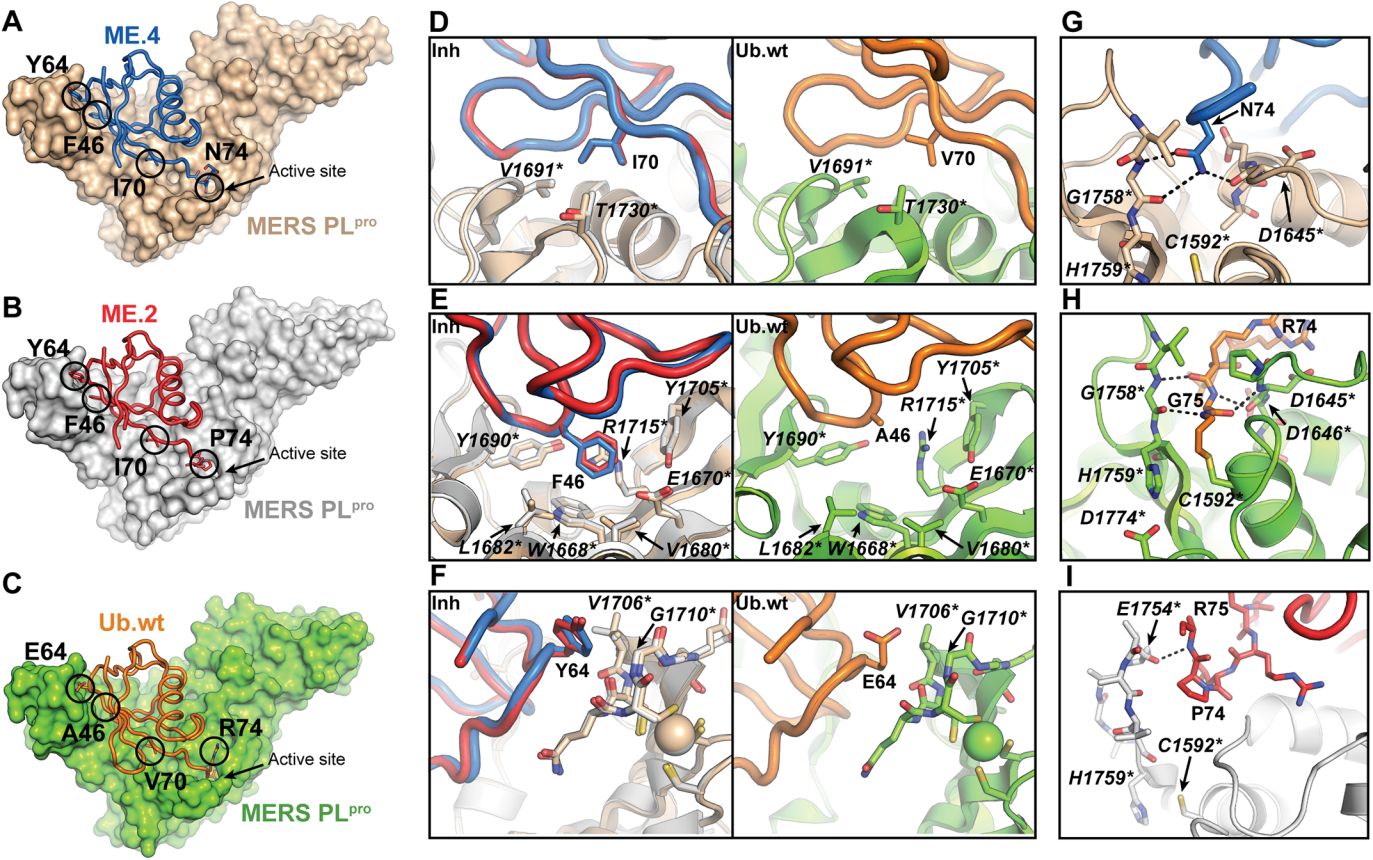
Structural basis for UbV inhibition of MERS-CoV PL^pro^. (**A-C**) Crystal structure of (**A**) the MERS-CoV PL^pro^-ME.4 complex, (**B**) the MERS-CoV PL^pro^-ME.2 complex, and (**C**) the MERS-CoV PL^pro^-Ub.wt complex (PDB ID: 4RF0). PL^pro^ domains are shown as surface representations, and coloured in wheat, gray and chartreuse for the PL^pro^-ME.4, -ME.2 and–Ub.wt complexes, respectively. ME.4, ME.2 and Ub.wt are shown as tubes and coloured in marine, red and orange, respectively. (**D**) Close up of a superposition of the MERS-CoV PL^pro^-ME.4 and -ME.2 complexes (left panel) showing detailed interactions between PL^pro^ and residue Ile^70^ of ME.4 or ME.2, and a comparison (right panel) of the same region in the PL^pro^-Ub.wt complex. PL^pro^ residues are shown as sticks and labeled with in italics with asterisks. (**E**) Close up of the MERS-CoV PL^pro^-ME.4 and -ME.2 complexes (left panel) showing detailed interactions between PL^pro^ and residue Phe^46^ of ME.4 or ME.2, and a comparison (right panel) of the same region in the PL^pro^-Ub.wt complex. (**F**) Close up of the MERS-CoV PL^pro^-ME.4 and -ME.2 complexes (left panel) showing detailed interactions between PL^pro^ and residue Tyr^64^ of ME.4 or ME.2, and a comparison (right panel) of the same region in the PL^pro^-Ub.wt complex. (**G**) Close up of ME.4 residue Asn^74^ bound near the active site of PL^pro^. Hydrogen bonds are represented by dashed black lines. (**H**) Close up of the C-terminus of Ub.wt covalently bound in the active site of PL^pro^. (**I**) Close up of ME.2 residue Pro^74^ bound near the active site of PL^pro^. Figures were generated using PyMOL [61].

To reveal the molecular basis for the inhibition of MERS-CoV PL^pro^ by UbVs, crystal structures of the enzyme were determined bound to ME.2 or ME.4 (**Fig 2A and 2B and Table 1**). Both UbVs bound in nearly identical orientations as Ub.wt (**Fig 2C and S3A Fig**) with interface surface areas of ~1000 Å^2^ [9, 27]. Substitutions common to both ME.4 and ME.2 at positions 46, 64 and 70 (**Fig 1A**) were found to promote more favorable hydrophobic interactions with the enzyme relative to Ub.wt. In comparison to Ub.wt residue Val^70^, UbV residue Ile^70^ extends further into a hydrophobic pocket of PL^pro^ formed by residues Thr^1730*^ and Val^1691*^ (*asterisks* denote amino acid numbering of MERS-CoV polyprotein) (**Fig 2D**). UbV residue Phe^46^ inserts into a hydrophobic pocket formed by PL^pro^ residues Trp^1668*^, Glu^1670*^, Val^1680*^, Leu^1682*^, Tyr^1690*^ and Tyr^1705*^, and forms a cation-π interaction with Arg^1715*^ (**Fig 2E**). These extensive interactions are not formed at the PL^pro^-Ub.wt interface with the Ub.wt residue Ala^46^. In addition, UbV residue Tyr^64^ makes more extensive hydrophobic interactions with Val^1706*^ and Gly^1710*^, compared to Ub.wt residue Glu^64^ **(Fig 2F**). The UbVs also differ in their C-terminal residues at positions 74, 75 and 77 (**Fig 1A**). In ME.4, Asn^74^ extends into the active site and forms a hydrogen bonding network with Asp^1645*^ and Gly^1758*^ (**Fig 2G**), which mimics hydrogen bonds formed by the same region of MERS-CoV PL^pro^ with Ub.wt residues Arg^74^, Gly^75^ and Gly^76^ (**Fig 2H**). In ME.2, Pro^74^ cannot form an analogous hydrogen bonding network, which likely contributes to the decreased inhibitory potency of ME.2 compared to ME.4 (**Fig 1D** and **S2A and S2B Fig**). Instead, Pro^74^ and the C-terminal tail are excluded from the active site and the main-chains interact through a hydrogen bond formed between the main-chain amide nitrogen of Arg^75^ and the backbone carbonyl of Glu^1754*^ (**Fig 2I**). A structural comparison of the C-terminal regions of ME.2 and ME.4 near the PL^pro^ active site is shown in **S4 Fig**. Additional substitutions in the N-terminal region of ME.4 and ME.2 (**Fig 1A, region 1**) do not make favourable contacts with MERS-CoV PL^pro^, and in fact, residues 8–10 and 7–10 of ME.4 and ME.2, respectively, failed to resolve in electron density maps (**S5 Fig**). Taken together, our structural analyses revealed that a small subset of substitutions is sufficient to enhance hydrophobic packing and hydrogen bond networks to endow ME.4 and ME.2 with highly potent and specific inhibitory activity against MERS-CoV PL^pro^.

**Table 1:** Crystallographic and refinement statistics for MERS-CoV PL^pro^ and CCHFV OTU bound to UbVs.

Crystal	MERS-CoV PL^pro^-ME.4	MERS-CoV PL^pro^-ME.2	CCHFV OTU-CC.4	CCHFV OTU-CC.2
**X-ray source**	Rigaku R-AXIS IV++	CLS 08B1-1	CLS 08ID-1	CLS 08ID-1
Crystal geometry				
Space group	C222_1_	C222_1_	P4_3_2_1_2	C2
Unit cell (Å)	*a* = 48.21 *b* = 110.96 *c* = 185.90; α = β = γ = 90°	*a* = 47.07 *b* = 109.78 *c* = 183.96; α = β = γ = 90°	*a = b* = 64.31 c = 277.58 α = β = γ = 90°	*a* = 101.4, *b* = 33.60 *c* = 71.25 *a = c* = 90°, *β* = 96.51°
**Crystallographic data**				
Wavelength (Å)	1.5419	1.2811	0.979	0.979
Resolution range (Å)	47.64–2.55 (2.64–2.55) *	45.97–2.70 (2.80–2.70)	47.17–2.10 (2.10–2.16)	43.43–1.50 (1.53–1.50)
Total observations	53264 (5257)	57650 (5719)	209641 (17075)	141907 (6821)
Unique reflections	16565 (1629)	13337 (1291)	34979 (2828)	38017 (1876)
Multiplicity	3.2 (3.2)	4.3 (4.4)	6.0 (6.0)	3.7 (3.6)
Completeness (%)	99.0 (100)	98.7 (97.6)	99.5 (99.8)	98.7 (96.6)
*R*_merge_	0.11 (0.63)	0.073 (0.68)	0.084 (0.89)	0.03 (0.62)
CC1/2	0.99 (0.68)	0.99 (0.88)	0.998 (0.926)	0.99 (0.79)
I/σI	10.9 (1.80)	16.69 (2.34)	12.2 (2.2)	18.7 (2.1)
Wilson B-factor (Å^2^)	37.21	54.23	33.96	18.63
**Refinement statistics**				
Reflections in test set	1656 (163)	1267 (99)	1998 (197)	1999 (195)
Protein atoms	3042	2976	3878	1926
Zinc atoms	1	1		
Solvent molecules	97	20	212	223
*R*_work_/*R*_free_	0.1972 / 0.2455	0.2169 / 0.2732	0.1743 / 0.2291	0.1563 / 0.1775
RMSDs				
Bond lengths/angles (Å/°)	0.002/0.49	0.001/0.41	0.010/1.04	0.008/0.97
Ramachandran plot				
Favored/allowed (%)	96/4	95/4.1	98/2	100/0
Average B factor (Å^2^)	38.16	57.52	40.59	28.27
Macromolecules	38.22	57.36	40.44	26.98
Solvent	36.74	52.53	43.57	38.29

*Values in parentheses refer to the highest resolution shell

**Fig 3:**
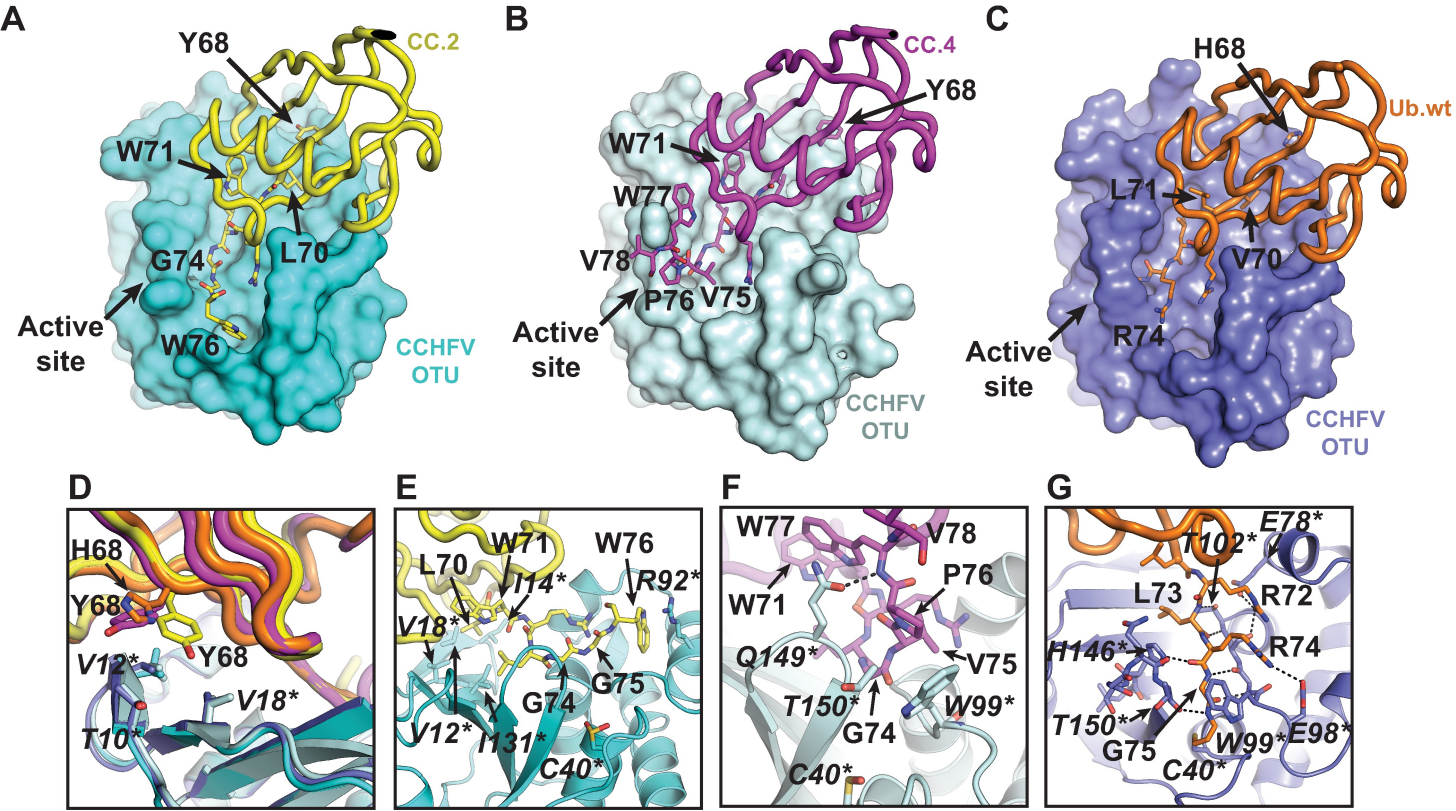
Structural basis for UbV inhibition of CCHFV OTU. (**A-C**) Crystal structure of (A) the CCHFV OTU-CC.2 complex, (**B**) the CCHFV OTU-CC.4 complex, and (**C**) the CCHFV OTU-Ub.wt complex (PDB ID: 3PT2). OTU domains are shown as surface representations, and coloured in cyan, light cyan and slate for the OTU-CC.2, -CC.4 and–Ub.wt complexes, respectively. CC.2, CC.4 and Ub.wt are shown as tubes and coloured in yellow, magenta and orange, respectively. (**D**) Overlay of the CCHFV OTU-CC.2, CC.4 and–Ub.wt structures showing interactions between CCHFV OTU and CC.2 or CC.4 residue Tyr^68^ or Ub.wt residue His^68^. UbV and Ub.wt residues are shown as sticks and labeled in regular font. CCHFV OTU residues are shown as sticks and labeled in italics with asterisks. (**E**) Close up of interactions between the C-terminus of CC.2 and CCHFV OTU. (**F**) Close up of interactions between the C-terminus of CC.4 and CCHFV OTU. (**G**) Close up of interactions between the C-terminus of Ub.wt and the active site of CCHFV OTU. Figures were generated using PyMOL [61].

Similarly, crystal structures of CCHFV OTU were determined bound to CC.2 or CC.4 to gain insight into how they selectively block the DUB and deISGylating activities of the viral protease (**Fig 3A and 3B** and **Table 1**). CC.2 and CC.4 were bound in the same orientation as Ub.wt with similar buried surface areas of ~1000 Å^2^ (**Fig 3C** and **S3B Fig**) [20, 21]. Interestingly, substitutions in these UbVs were concentrated in the C-terminal region (**Fig 1A**) and only substitutions at position 68 and downstream were found to interact with the enzyme. In both UbVs, Tyr^68^ improves hydrophobic packing with Thr^10*^, Val^12*^ and Val^18*^ (*asterisks* denote amino acid numbering of the large segment-encoded protein of CCHFV), relative to His^68^ in Ub.wt (**Fig 3D**). In CC.2, residue Leu^70^ projects further into a hydrophobic cavity in CCHFV OTU formed by residues Val^12*^, Ile^14*^, Val^18*^ and Ile^131*^, than does the equivalent Ub.wt residue Val^70^ (**Fig 3E**). The conformational freedom of a Gly substitution at position 74 enables the C-terminal tail of each UbV to form numerous favorable interactions with the enzyme. Residues Gly^75^ and Val^75^ of CC.2 and CC.4, respectively, occupy space on the CCHFV OTU surface, which in the case of Ub.wt is occupied by the side-chain of Arg^74^ (**Fig 3E–3G**). This alternative conformation permitted by Gly^74^ in CC.2 and CC.4 allows the side-chain of Trp^76^ or Trp^77^ of CC.2 or CC.4, respectively, to pack within different adjacent grooves near the CCHFV OTU active site, with Trp^76^ of CC.2 forming a cation-π interaction with Arg^92*^ (**Fig 3E**), and the amide nitrogen group of CC.4 residue Trp^77^ forming a hydrogen bond with the side chain of Gln^149*^ (**Fig 3F**). Conversely, Trp^71^ in both CC.2 and CC.4 does not interact with CCHFV OTU, but instead packs into a hydrophobic cavity within each UbV (**Fig 3E and 3F**). Differences between the orientations of the C-terminal tails of CC.2 and CC.4 arise from the variation at positions 75 and 76. In CC.4, residue Val^75^ packs against CCHFV OTU residue Trp^99*^, and Pro^76^ appears to restrict conformational freedom and enable hydrophobic interactions with Trp^99*^ and Thr^150*^ (**Fig 3F**). Together with the packing orientation of Trp^77^, these additional contacts likely account for the high binding affinity of CC.4 for CCHFV OTU (**S2 Table**).

### UbVs inhibit MERS-CoV PL^pro^ activity in cell culture assays

**Fig 4:**
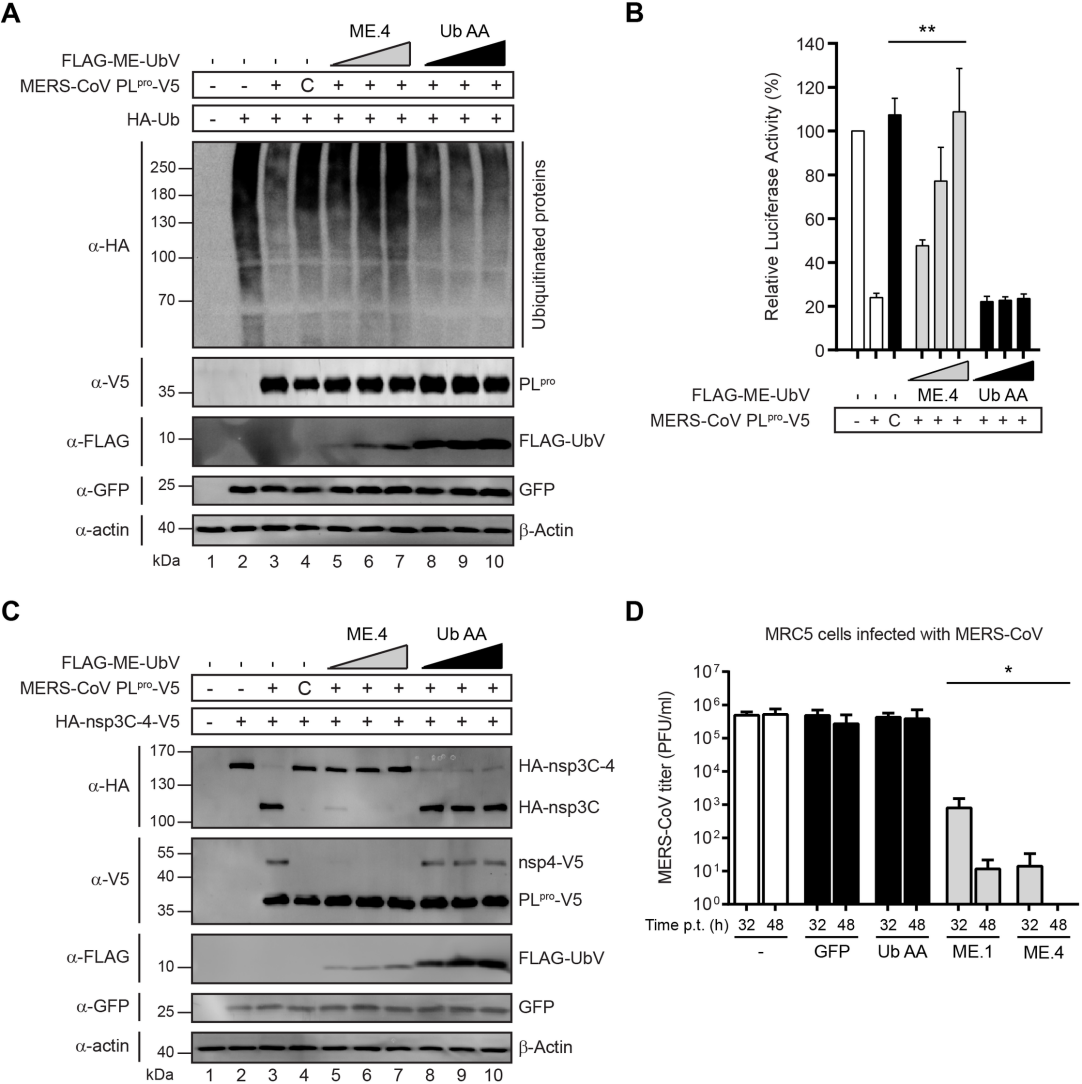
UbVs inhibit proteolytic activity of MERS-CoV PL^pro^ in cell culture and affect MERS-CoV replication. (**A**) The effects of UbVs on the DUB activity of MERS-CoV PL^pro^ was determined by co-transfecting HEK293T cells with plasmids encoding HA-Ub, MERS-CoV PL^pro^-V5 (wild type or the active site mutant C1592A annotated as “C” throughout the rest of the figure), FLAG-ME-UbV as indicated (in increasing dose) and GFP (as a transfection control). Cells were lysed 18 hours post transfection and expressed proteins were analyzed by western blotting. DUB activity of MERS-CoV PL^pro^ was visualized by the deconjugation of HA-Ub from cellular proteins. (**B**) Assessment of the inhibitory effects of UbVs on the suppression of the IFN-β promoter by MERS-CoV PL^pro^. HEK293T cells were transfected with plasmids encoding firefly luciferase reporter gene under control of the IFN-β promoter, *Renilla* luciferase, innate immune response inducer mitochondrial antiviral signaling protein (MAVS), MERS-CoV PL^pro^-V5 (wild type or the active site mutant C) and FLAG-tagged UbVs (in increasing dose). Cells were lysed 16 hours post transfection and both firefly and *Renilla* luciferase activities were measured. Results represent at least three independent experiments. Significance relative to wild-type without expression of a UbV was calculated using an unpaired two-tailed Student’s *t* test and significant values were indicated: ** *p* < 0.01. *Bars* represent mean and *error bars* represent S.D. (**C**) Proteolytic cleavage capability of MERS-CoV PL^pro^ was assessed in the presence of the UbVs. N-terminally HA-tagged and C-terminally V5-tagged nsp3C-4 (excluding the PL^pro^ domain) was co-expressed with V5-tagged MERS-CoV PL^pro^-V5 (wild type or the active site mutant C), FLAG-ME-UbV (with increasing doses) and GFP (as a transfection control). Cells were lysed 18 hours post transfection and proteolytic cleavage activity was assessed by western blotting to detect generation of N-terminal HA-tagged nsp3C and C-terminal V5-tagged nsp4 cleavage products. (**D**) MERS-CoV titers of collected supernatants from lentivirus transduced and, subsequently, MERS-CoV infected MRC5 cells. MRC-5 cells were transduced with lentiviruses encoding FLAG-UbVs, FLAG-Ub.AA or GFP (latter two as controls) and, either 32 hours or 48 hours post-transduction, the cells were infected with MERS-CoV at a multiplicity of infection of 0.01. After another 32 hours, culture supernatants were harvested and MERS-CoV titers were determined by plaque assays on Vero cells. Significant difference relative to MERS-CoV titers from lentivirus transduced MRC5 cells expressing Ub.AA is indicated: * *p*<0.05. *Bars* represent mean and *error bars* represent S.D.

To explore the effects of UbVs on MERS-CoV PL^pro^ activity in cells, deubiquitination assays were performed by transfecting cells with combinations of plasmids encoding the following proteins: HA-tagged Ub (which becomes conjugated to cellular proteins), MERS-CoV PL^pro^, and UbVs (which are unconjugatable due to substitutions in the C-terminal di-Gly motif). A clear decrease of cellular HA-Ub conjugates was observed during co-expression of MERS-CoV PL^pro^, while there was no effect upon expression of a catalytically inactive mutant (**Fig 4A,** compare lanes 3 and 4). The co-expression of increasing doses of different UbVs attenuated MERS-CoV PL^pro^ DUB activity to varying degrees (**Fig 4A and S6A Fig**). In a dose-dependent manner, ME.4 co-expression resulted in severe inhibition of HA-Ub deconjugation mediated by MERS-CoV PL^pro^, whereas co-expression of an unconjugatable form of Ub.wt as a negative control (Ub.AA, which contains Gly75Ala and Gly76Ala substitutions) had no effect on the DUB activity of MERS-CoV PL^pro^ at any dose (**Fig 4A,** compare lanes 5–7 to 8–10). Like ME.4, ME.2 had a strong effect resulting in near complete inhibition of MERS-CoV PL^pro^ DUB activity at the lowest UbV dose, whereas the inhibitory effect of ME.1 and ME.3 was apparent only at higher UbV doses (**S6A Fig**). Notably, none of the UbVs inhibited the DUB activity of the closely related SARS-CoV PL^pro^, highlighting their specificity for MERS-CoV PL^pro^ (**S7A Fig**). A superposition of ME.4 onto the Ub domain of a previously determined SARS-CoV-Ub complex suggests that Phe^46^ and Ile^70^ of ME.4 clash with Val^188*^ and Met^209*^ of SARS-CoV PL^pro^, respectively, and offers a plausible explanation for the specificity of ME.2 and ME.4 toward MERS-COV PL^pro^ (**S8 Fig**).

We previously found that the DUB activity of MERS-CoV PL^pro^ suppresses IFN-β promoter activity upon activation of cellular innate immune signaling [9]. In a luciferase-based IFN-β reporter assay we show that ectopically expressed UbVs competed with endogenous Ub for binding to MERS-CoV PL^pro^ resulting in an alleviated suppression of the IFN-β promoter activity (**Fig 4B and S6B Fig**). Consistent with described binding and inhibition data, ME.2 and ME.4 were more potent than ME.1 and ME.3 at blocking the ability of MERS-CoV PL^pro^ to suppress IFN-β promoter activation, whereas none of the UbVs were able to block suppression of the IFN-β promoter activity by SARS-CoV PL^pro^ (**S7B Fig**). The UbVs thus prevented MERS-CoV PL^pro^-mediated suppression of cellular anti-viral innate immune responses, and in a remarkably selective, virus-specific manner.

A critical step in the replication cycle of MERS-CoV is the processing of viral polyproteins into functional non-structural proteins (nsps) that is accomplished in part by the protease activity of PL^pro^, which cleaves the nsp1↓2, nsp2↓3, and nsp3↓4 junctions [28]. In order to assess the ability of UbVs to inhibit MERS-CoV PL^pro^-mediated polyprotein processing activity, an *in trans* cleavage assay was performed [9]. FLAG-tagged UbVs and V5-tagged MERS-CoV PL^pro^ were co-expressed with N-terminally HA-tagged and C-terminally V5-tagged nsp3C-4 (HA-nsp3C-4-V5), a fragment of the viral polyprotein encompassing the C-terminal part of nsp3 (excluding the PL^pro^ domain) and nsp4. *In trans* cleavage of the nsp3↓4 junction is indicative of proteolytic activity of PL^pro^ during infection [9]. MERS-CoV PL^pro^ efficiently cleaved HA-nsp3C-4-V5 into HA-nsp3C and nsp4-V5 products, whereas the active site mutant did not (**Fig 4C,** compare lanes 3 and 4). The cleavage of the nsp3↓4 site was not affected upon expression of the negative control Ub.AA, whereas only a fraction of HA-nsp3C-4-V5 was cleaved upon expression of ME.4 or ME.2 at the lowest dose, and cleavage was completely blocked at higher UbV doses (**Fig 4C** compare lanes 5–7 to 8–10, **S6C Fig**). Increasing doses of ME.1 and ME.3 also resulted in reduced cleavage as gradually more HA-nsp3C-4-V5 precursor was observed (**S6C Fig**).

### UbVs block MERS-CoV replication in cells

To directly asses the ability of UbVs to inhibit MERS-CoV replication, MERS-CoV PL^pro^-specific UbVs were ectopically expressed in cell culture, and cells were subsequently infected with MERS-CoV. MRC5 and HuH-7 cell lines were transduced with lentiviruses encoding FLAG-tagged UbVs, Ub.AA, or GFP. Efficient expression of FLAG-ME.1 and GFP in these cells was confirmed by fluorescence microscopy and by western blotting (**S9 Fig**). Either 32 or 48 hours post-transduction, cells were infected with MERS-CoV at a multiplicity of infection of 0.01, and MERS-CoV titers were determined from supernatants harvested 32 hours post infection (**Fig 4D**). In MRC5 cells, ME.1 and ME.4 expression resulted in significantly lower virus titers as these dropped from 5 x 10^5^ plaque forming units (PFU)/ml recovered from control cells to 1,000 or 10 PFU/ml, respectively, when the MERS-CoV infection was started 32 hours post-transduction (**Fig 4D**). The effect of the UbVs was even more pronounced in MRC5 cells that were infected with MERS-CoV 48 hours post-transduction, as virus titers dropped below 10 PFU/ml upon expression of ME.4, which represented a reduction in infectious progeny titers of more than four orders of magnitude (**Fig 4D**) and correlated with higher expression of the UbVs at this time point (**S9 Fig and S10 Fig**). In HuH-7 cells the expression of GFP or Ub.AA did not affect MERS-CoV titers compared to the non-transduced cells, whereas ME.1 expression led to a two orders of magnitude reduction in virus titer, and an even greater reduction of more than three orders of magnitude was observed upon ME.4 expression (**S11 Fig**). The effect of UbVs on MERS-CoV progeny titers was more severe in MRC5 cells compared to HuH-7 cells, which might be due to generally higher expression of UbVs in MRC5 cells than in HuH-7 cells (**Fig 4D, S9B Fig and S11 Fig**). Taken together, these studies show that UbVs readily inhibit the proteolytic activities of MERS-CoV PL^pro^ in cells and provide extremely effective protection from MERS-CoV infection.

## Discussion

The continued introduction of viruses from zoonotic sources into human populations poses a serious and constant threat to human health [8]. In the case of coronavirus infection, therapeutic options are limited, and vaccine development remains in progress [8, 29]. Here, we describe a unique protein engineering platform that can be used to rapidly generate UbVs to selectively block the activity of vDUBs from a range of evolutionarily distinct viral lineages. Indeed, UbVs generated against MERS-CoV almost completely abolished the replication of this virus in host cells. Meanwhile, none of the UbVs generated in this study likely cross-reacted with human DUBs as no toxicity was observed upon expression of UbVs, demonstrating their potential therapeutic safety.

Targeting intracellular targets with protein-based inhibitors is currently not a therapeutic option due to the practical limitations of immunogenicity and the lack of efficient means for delivering proteins in to cells *in vivo*. However, progress continues to be made on methods for intracellular delivery of mRNA or proteins [30–32], and strategies to reduce immunogenicity, including the use of mirror-image proteins [33], have been developed. Thus, in the future, we are hopeful that UbVs may become bona fide drug candidates, but at present, they are best viewed as tool compounds that can enable drug discovery. For example, while the interface between the UbVs and vDUBs is large, we have recently used combinatorial mutagenesis to reveal a smaller site on human USPs within the UbV interaction interface, which may be amenable as a target for small-molecule inhibitor design [34]. Indeed, while the design of small molecules that target protein-protein interfaces remains challenging, there are successful examples where polypeptide-based tools have facilitated design and screening of small molecule inhibitors [35].

A plethora of viruses causing human disease encode for vDUBs implicated in replication and/or pathogenesis, emphasizing the potential clinical importance of vDUBs as therapeutic targets [9, 36–41]. The UbV development platform can also be readily extended to target viruses that infect plants and animals of economic importance, including porcine reproductive and respiratory syndrome virus, equine arteritis virus, bovine coronavirus, transmissible gastroenteritis virus, porcine epidemic diarrhea virus and turnip yellow mosaic virus, all of which encode vDUBs that are essential for virus replication [22]. In contrast, some vDUBs, like the CCHFV OTU protease, do not appear to process viral polyproteins and are instead dedicated to increasing viral pathogenicity by suppressing cellular innate immunity [17, 42]. Interestingly, the CCHFV OTU protease comprises a domain on the viral polymerase, and targeting it with a UbV may not only suppress viral pathogenicity but RNA replication as well, since it has been found that viral OTU DUB activity can suppress proteasome-dependent viral polymerase degradation [43]. Additionally, the strong binding of a UbV to the viral polymerase could cause steric hindrance and in this way affect polymerase function.

With current transgenic technologies [44], livestock and plant crops expressing virus-specific UbVs could be readily engineered to generate virus-resistant organisms. Significant progress has also been made towards the development of animal models of MERS-CoV infection ([45–49] and reviewed in [50]), which includes mouse models used in concert with mouse-adapted MERS-CoV strains, together mimicking MERS-CoV pathology observed in humans [51]. These technologies will provide attractive platforms to assess the efficacy of UbV delivery systems to treat MERS-CoV infection *in vivo*.

The utilization of the Ub scaffold as a template for vDUB inhibitor development exploits the large Ub-binding interface to provide a high degree of specificity in comparison to small molecule-based approaches, since the latter usually rely on targeting the active site of the viral protease, thereby invoking the risk of cross-reactivity with cellular proteases. The amino acid substitutions in UbVs that confer potent inhibition of vDUBs are distributed across relatively large binding interfaces, and we speculate that resistance to UbVs is less likely to develop in comparison to small-molecule inhibitors. The larger binding surface and the much tighter binding affinities of UbVs (low to sub-nanomolar, **Fig 1C**) compared to reported micromolar affinities for small-molecule inhibitors of MERS-CoV PL^pro^ [52] imply that rather extensive mutations in the viral protease will be necessary to efficiently repel the UbV. Since development of resistance mutations requires virus replication, the extreme reduction in MERS-CoV progeny titers upon expression of UbVs during infection is therefore expected to severely delay occurrence of resistance. Additionally, if a resistant virus happens to be already present in the quasi species it is likely that mutations to repel the UbV from the vDUB will concomitantly lead to less optimal binding of the viral protease to Ub/ISG15 itself and maybe even the virus replicase polyproteins. Therefore, the resistant virus is expected to be attenuated and to have at least reduced deubiquitinating/deISGylating activity that is important for the virus’ capacity to suppress host innate immune responses [9, 53]. This idea is supported by described escape mutants for MHV to a broad-spectrum CoV 3C-like protease inhibitor where the inhibitor-resistant virus was attenuated in mice, highlighting that the mutant was generated at the cost of replicative fitness [54] In future experiments we will experimentally assess the development of UbV resistance during infection in cell culture and mouse models, which will show whether our speculations are correct.

Ultimately, in the event that resistant strains do emerge in a clinical setting, new UbVs targeting these strains can also be generated rapidly. Most importantly, unlike small-molecule approaches that can take years to implement and often fail, phage display yields potent and selective viral inhibitors in weeks, a rate that could allow therapeutic development to keep pace with the continued emergence of pathogenic viruses and limit their pandemic potential. Together, these findings make further exploration of UbVs as potent and rapidly developed antiviral agents an exciting and promising venture.

## Materials and methods

### Selection of ubiquitin variants

The phage-displayed UbV library used in this study was re-amplified from Library 2 as previously described [25]. Protein immobilization and the following UbV selections were done according to established protocols [26, 55]. Briefly, purified viral proteases were coated on 96-well MaxiSorp plates (Thermo Scientific 12565135) by adding 100 μL of 1 μM proteins and incubating overnight at 4°C. Afterwards, five rounds of selections using the phage-displayed UbV library were performed against immobilized proteins including the following steps: (*a*) Each phage particle in the library pool displays a unique UbV and encapsulates the encoding DNA; (*b*) Binding phages are captured with an immobilized protein; (*c*) Non-binding phages are washed away; and (d) Bound phages are amplified by infection of bacteria. The enriched phage pool is cycled through additional rounds of selection to further enrich for protein-binding UbVs. After the fifth round of binding selections, individual phages with improved binding properties were identified by phage ELISA using established techniques and subjected to DNA sequencing of the phagemids to obtain UbV sequences [26, 55].

### Protein crystallization

#### 1. MERS-CoV PL_pro_-ME.2 and -ME.4 complexes

To form the non-covalent MERS-CoV PL^pro^-UbV complexes, a 4-fold molar excess of ME.4 or ME.2 was incubated with MERS-CoV PL^pro^ overnight at 4°C. The excess, unbound UbVs were removed from the sample using a Superdex 75 size exclusion column and fractions containing the MERS-CoV PL^pro^-UbV complex were pooled and concentrated to 10 mg/mL. The MERS-CoV PL^pro^-ME.4 complex was found to crystallize under similar conditions to those previously reported for the MERS-CoV PL^pro^-Ub complex [9], with optimal crystals appearing in 0.1 M trisodium citrate pH 5.6, 20% (w/v) polyethylene glycol (PEG) 4000 and 20% (v/v) isopropanol. Crystals of the MERS-CoV PL^pro^-ME.2 complex were grown in 0.1 M trisodium citrate pH 5.6, 19% (w/v) PEG 4000 and 19% (v/v) 1,2-isopropanediol. Crystals were grown by mixing PL^pro^-UbV (10 mg/mL and 9 mg/mL for PL^pro^-ME.4 and PL^pro^-ME.2, respectively) with crystallization solution at a 1:1 volumetric ratio (2 μL MERS-CoV PL^pro^-UbV + 2 μL well solution). Immediately prior to mixing, 1 M DTT was added to the MERS-CoV PL^pro^-UbV complexes to a final concentration of 10 mM to prevent oxidation of the sample.

#### 2. CCHFV OTU_169_-CC.2 and CCHFV OTU_185_-CC.4 complexes

Purified CCHFV OTU was pooled with 2-3-fold molar excess of purified UbV and dialyzed overnight against 50 mM Tris pH 8.0, 150 mM NaCl and 2 mM DTT. Protein complexes were concentrated and loaded onto a Superdex 75 size exclusion column and eluted in 50 mM Tris, 150 mM NaCl and 2 mM DTT. For all samples, a single peak corresponding to the respective complex was observed in the gel filtration profile and two bands corresponding to the CCHFV OTU and respective UbV were observed by SDS-PAGE, indicating the high purity of the complexes. The CCHFV OTU_169_-CC.2 complex was concentrated to 12 mg/ml for crystallization trials, and initial crystals and crystalline material obtained from preliminary screens were used to prepare seed stocks for microseed matrix screening [56, 57], which was set up for the hanging drop vapor diffusion method in 48-well VDX plates (Hampton Research) and carried out using conventional screens (Qiagen) at 4°C, with and without heterogeneous nucleation using 0.3–0.4 cm strands of human hair [58]. Total drop volume was 2 μl containing equal volumes of the protein complex and the well solution. Crystals of the CCHFV OTU_169_-CC.2 complex were grown in 30% (w/v) PEG 4000, 0.2 M CaCl_2_ and 0.1 M HEPES pH 7.5 and appeared after 5–8 days.

The CCHFV OTU_185_-CC.4 complex was concentrated to 23 mg/ml and initial leads were observed with a combinatorial approach using microseed matrix screening with crystallization screens (Qiagen) along with the Silver Bullets screen (Hampton Research) and micro-seeding using crystals of a CCHFV OTU_185_-CC.5 (a weaker binding variant selected by phage display) complex. Using the hanging drop vapor diffusion method and 48-well VDX trays (Hampton Research), screens were set up at 20°C with a reservoir volume of 150 μl, and a drop size of 3.5 μl, which comprised of 1.5 μl of the protein complex, 1 μl of the reservoir solution and 1 μl of Silver Bullet additive, added in this order. Crystals were obtained with 25% (w/v) PEG 3350, 0.1 M Tris pH 7.0 and 0.2 M sodium chloride. The Silver Bullet formulation in the drop was as follows: 0.16% (w/v) each of 5-Sulfosalicylic acid dehydrate, dodecanedioic acid, hippuric acid, mellitic acid, oxalacetic acid, suberic acid and 0.02 M HEPES sodium pH 6.8.

Data collection procedures are described in Supporting Information.

### Lentivirus transduction and MERS-CoV infections

To produce lentiviruses, HEK293T cells (Virgin lab, Washington University School of Medicine) grown in a T175 flask were transfected with packaging vectors pMDLg/pRRE and pRSV-REV, envelope protein-expressing vector pCMV-VSVG [59] and the transfer vector (pLenti6.3/TO/V5-DEST containing GFP or FLAG-UbVs) using polyethylenimine (PEI; Polysciences Inc.). Medium was replaced 24 h post transfection and 48 h and 72 h post transfection supernatant was collected, centrifuged (1000 x g for 10 min) and filtered through a 0.45 μm filter before storage at -80°C. Titers of the lentivirus particles were determined by p24 antigen ELISA (ZeptoMetrix). HuH-7 (Bartenschlager lab, Heidelberg University) and MRC5 cells (CCL-171; American Type Culture Collection) grown in a 12-well plate were transduced with lentiviruses encoding GFP or FLAG-ME.1 diluted in DMEM containing 2% FCS and 8 μg/ml Polybrene (Sigma Aldrich). Medium was replaced 24 h post transduction (pt), and 32 h or 48 h pt protein lysates were obtained by adding 250 μl 2xLSB containing 25 mM NEM to each well while cells grown on coverslips were fixed with 3% paraformaldehyde (PFA) in PBS.

GFP and FLAG-ME.1 expression were analyzed by Western blotting as described in the supporting information. Fixed cells that were grown on coverslips were permeabilized with 0.1% Triton X-100 in PBS and subsequently indirect immunofluorescence assays were carried out. Primary and secondary antibodies were diluted in PBS containing 5% FCS and Hoechst 33258 was added to the secondary antibody dilution to stain nuclear DNA. Coverslips were analyzed with a Zeiss Axioskop 2 fluorescence microscope with an Axiocam HRc camera.

To obtain a cell population in which >90% of the cells expressed GFP, the amount of lentivirus yielding 120 ng of p24 was required to transduce 1x10^5^ HuH-7 cells and 40 ng of p24 for 1x10^5^ MRC5 cells was required. HuH-7 or MRC5 cells (1x10^5^/12-well) were transduced with lentiviruses encoding GFP, FLAG-Ub.AA, FLAG-ME.1 or FLAG-ME.4. Cells were infected with MERS-CoV with a multiplicity of infection of 0.01 32 h or 48 h pt. MERS-CoV inocula were prepared in PBS containing 50 μg/ml DEAE-dextran and 2% FCS. Cells were inoculated for 1 h at 37°C and the inoculum was replaced with EMEM containing 2% FCS. Supernatants were harvested 32 h post MERS-CoV infection and simultaneously cells were lysed in 4x LSB for Western blotting analysis. MERS-CoV titers were determined by plaque assays on Vero cells (Department of Viroscience, Erasmus Medical Center) as described by van den Worm et al. [60]. MERS-CoV infection experiments were performed at least twice and plaque assays were performed in duplicate in order to determine MERS-CoV titers. Work with MERS-CoV was performed inside biosafety cabinets in Biosafety Level 3 facilities at Leiden University Medical Center.

Full details of materials and methods are described in Supplemental files (S1 Appendix).

### Accession numbers

Coordinate files and structure factors have been deposited to the Protein Data Bank under accession codes 5V6A and 5V69 for the MERS-CoV PL^pro^-ME.2 and–ME.4 structures, respectively, and 5V5H and 5V5G for the CCHFV OTU-CC.2 and–CC.4 structures, respectively.

## Supporting information

S1 Appendix Supplemental materials and methods. (DOCX)

S1 Fig UbVs bound with high affinity to MERS-CoV PL^pro^ and CCHFV OTU. (**A**) Binding curves of UbVs to the cognate viral proteases (left panel: MERS-CoV PL^pro^; right panel: CCHFV OTU), measured by ELISA. The half maximal binding concentrations (EC_50_) of UbVs to indicated vDUBs were determined by established methods [26] and are listed in **S1 Table**. Viral proteases (1 μM) were immobilized in microtiter plates. Serial dilutions of FLAG-tagged UbV or Ub (up to 4 μM, 24 points) were added and incubated for 20 min at room temperature. Wells were washed and bound UbV/Ub was detected by anti-FLAG-HRP conjugate antibody and colorimetric development of TMB peroxidase substrate. The absorbance at 450 nm (y-axis) was plotted against Log (UbV/Ub concentration, nM) (x-axis). Data were presented as the mean ± SD (N = 3). (**B**) Binding curves of wild type Ub (Ub.wt) to MERS-CoV PL^pro^ (left) and CCHFV OTU (right). Experiments were performed as described in (**A**) except the concentration of Ub was increased (up to 10 mM, 24 serial dilutions). Data were presented as the mean ± SD (N = 3).
(TIF)

S2 Fig MERS-CoV PL^pro^ and CCHFV OTU are inhibited by UbVs in vitro. (**A-B**) Inhibition of MERS-CoV PL^pro^ (left) or CCHFV OTU (right) by the cognate UbVs shown as dose-response curves using Ub-AMC (**A**) or ISG15-AMC (**B**) as a substrate. The IC_50_ values were determined as the concentrations of UbVs that reduced deubiquitination or deISGylation activity by 50% (**S1 Table**). The wt Ub data obtained in the deISGylation assay can not be fitted by GraphPad Prism so no lines were shown.
(TIF)

S3 Fig MERS-CoV- and CCHFV-specific UbVs bind their cognate DUBs in comparable orientations to Ub.wt. (**A**) Superposition of the MERS-CoV PL^pro^-Ub.wt, -ME.2 and -ME.4 complexes. PL^pro^ is displayed as ribbons, and coloured in chartreuse, gray and wheat in the PL^pro^-Ub.wt, -ME.2 and -ME.4 structures, respectively. The Ub and UbV structures are displayed as tubes, and coloured in orange, red and marine in the PL^pro^-Ub.wt, -ME.2 and -ME.4 structures, respectively. (**B**) Superposition of the CCHFV OTU-Ub.wt, -CC.2 and CC.4 complexes. CCHFV OTU is displayed as ribbons, and coloured in slate, cyan and pale cyan in the CCHFV OTU-Ub.wt, -CC.2 and -CC.4 structures, respectively. The Ub and UbV structures are displayed as tubes, and coloured in orange, yellow and magenta in the CCHFV OTU-Ub.wt, -CC.2 and -CC.4 structures, respectively. Structures were aligned within PyMOL [61].
(TIF)

S4 Fig Comparison of the C-terminal regions of ME.2 and ME.4 in the active site of MERS-CoV PL^pro^. (**A**) Superposition of the C-terminal regions of the MERS-CoV PL^pro^-ME.2 and–ME.4 structures. PL^pro^ is coloured in gray and wheat in the MERS-CoV PL^pro^-ME.2 and–ME.4 structures, and ME.2 and ME.4 are coloured in red and marine, respectively. PL^pro^ active site residues His1759 and Cys1592 are shown as sticks, along with additional PL^pro^, ME.2 and ME.4 residues involved in binding. (**B**) Close up of the C-terminus of ME.4 in the MERS-CoV PL^pro^-ME.4 complex, with PL^pro^ depicted in a surface representation. (**C**) Close up of the C-terminus of ME.2 in the MERS-CoV PL^pro^-ME.2 complex, with PL^pro^ depicted in a surface representation.
(TIF)

S5 Fig Residues in the N-terminal β-hairpin of ME.4 and ME.2 are disordered. (**A**) Cartoon representation of ME.4 (marine). Dashed line indicates missing residues 8–10 which were not resolved in the electron density maps. A 2*F*_o_-*F*_c_ electron density map is displayed as blue mesh and contoured at 1.0 RMSD. (**B**) Cartoon representation of ME.2 (red). Dashed line indicates missing residues 7–10. Figure generated with PyMOL [61].
(TIF)

S6 Fig Proteolytic activity of MERS-CoV PL^pro^ is inhibited by UbVs. (**A**) Inhibition of MERS-CoV PL^pro^ DUB activity by ME.1, ME.2 and ME.3 was determined by expressing HA-Ub, MERS-CoV PL^pro^-V5 (wild type or the active site mutant C1592A designated as C), FLAG-ME-UbV (500, 750 or 1000 ng of the appropriate plasmid) and GFP (as a transfection control) in HEK293T cells. After obtaining protein lysates the expressed proteins were separated on a SDS-PAGE gel, blotted and visualized after antibody incubations. (**B**) Suppression of the IFN-β promoter activity by MERS-CoV PL^pro^ in the presence of UbVs was assessed by transfecting plasmids encoding firefly luciferase reporter gene under control of the IFN-β promoter, *Renilla* luciferase, MAVS, MERS-CoV PL^pro^-V5 (wild type or the active site mutant C) and FLAG-tagged UbVs (250, 500 or 750 ng). Firefly and *Renilla* luciferase activities were measured 16 h post transfection and significance relative to wild-type without expression of a UbV was calculated using an unpaired two-tailed Student’s *t* test. Significant values were indicated: ** *p* < 0.01. *Bars* represent mean and *error bars* represent S.D (N = 3). (**C**) Proteolytic cleavage capability of MERS-CoV PL^pro^ was assessed in the presence of the UbVs. N-terminally HA-tagged and C-terminally V5-tagged nsp3C-4 (a polyprotein fragment excluding PL^pro^) was co-expressed with MERS-CoV PL^pro^-V5 (wild type or the active site mutant C), FLAG-ME-UbV (at increasing concentrations) and GFP (as a transfection control). Cells were lysed 18 h post-transfection and expressed proteins were analyzed by Western blotting.
(TIF)

S7 Fig MERS-CoV-directed UbVs do not inhibit the DUB activity of SARS-CoV PL^pro^. (**A**) SARS-CoV PL^pro^’s DUB activity in the presence of UbVs was determined by co-transfecting HEK293T cells with plasmids encoding HA-Ub, SARS-CoV PL^pro^-V5 (wild type or the active site mutant C1651A designated as C), FLAG-ME-UbV (1000 ng) and GFP (as a transfection control). 18 h post-transfection cells were lysed and deconjugation of HA-tagged Ub by SARS-CoV PL^pro^ was visualized via Western blotting. (**B**) HEK293T cells were transfected with plasmids encoding firefly luciferase reporter gene under control of the IFN-β promoter, *Renilla* luciferase, MAVS, SARS-CoV PL^pro^-V5 (wild type or the active site mutant C; 100 ng) and FLAG-tagged UbVs (750 ng). Cells were lysed 16 h post-transfection and both firefly and *Renilla* luciferase activities were measured. Significance relative to wild-type without expression of a UbV was measured using an unpaired two-tailed Student’s *t* test; significant values were indicated: ** *p* < 0.01. *Bars* represent mean and *error bars* represent S.D (N = 3).
(TIF)

S8 Fig Structural model of the SARS-CoV PL^pro^ domain bound to the MERS-CoV PL^pro^-specific ME.4. (**A**) The SARS-CoV PL^pro^ domain is shown as a cartoon representation (yellow-orange). ME.4 and Ub.wt are shown in tubes (marine and orange, respectively). The ME.4 structure determined herein was superposed over Ub bound to the SARS-CoV PL^pro^ domain (4M0W [62]) (**B**) Close-up of residue clashes occurring between SARS-CoV PL^pro^ and ME.4. Residues are shown as spheres, with SARS-CoV PL^pro^ residues indicated with asterisks and in italics. SARS-CoV PL^pro^ residue M209 clashed with ME.4 residue I70, compared with Ub residue V70 (**C**). (**D**) SARS-CoV PL^pro^ residues E204 and V188 clash with ME.4 residues Q48 and V188, respectively, compared to Ub.wt residues K48 and A46 (**E**). Figure generated in PyMOL [61].
(TIF)

S9 Fig Analysis of lentivirus transduction of MRC5 and HuH-7 cells. (**A, B**) Western blot analysis of transduced MRC5 and HuH-7 cells with lentiviruses encoding GFP (**A**) or FLAG-ME.1 (**B**) both 32 h and 48 h pt. As a control cells were mock transduced (designated as M). Relative expression of GFP and FLAG-ME.1 was quantified and normalized to actin and expression levels in MRC5 cells 48 h pt were set at 100%. (**C**) GFP transduced MRC5 and HuH-7 cells were fixed 32 h or 48 h pt and nuclear DNA was stained using Hoechst. Images were taken using fixed exposure times for both the GFP and Hoechst signal. (**D**) Immunofluorescence assay of FLAG-ME.1-transduced MRC5 and HuH-7 cells that were fixed 32 h or 48 h pt. Cells were labelled with a mouse monoclonal antibody recognizing FLAG followed by labelling with a secondary Alexa488-conjugated goat anti-mouse antibody. Exposure times were kept the same for each image.
(TIF)

S10 Fig Western blot analysis of MERS-CoV infection on transduced MRC5 cells shows decreased viral protein production as a result of UbV expression. Upon collection of supernatants after MERS-CoV infection of transduced MRC5 cells protein lysates were obtained. Expression of two viral proteins was analyzed by Western blotting, MERS-CoV nsp4 (using cross-reacting SARS-CoV nsp4 antiserum), and MERS-CoV ORF4B. Lentivirus-induced expression of FLAG-UbVs or GFP was confirmed and actin was used as a loading control. Representative Western blots are shown for transduced MRC5 cells that were infected with MERS-CoV at a multiplicity of infection of 0.01 either 32 h pt (**A, B**) or 48 h pt (**C, D**).
(TIF)

S11 Fig Titers of MERS-CoV progeny decreased upon infection of HuH-7 cells expressing UbVs. (**A**) HuH-7 cells were transduced with lentiviruses encoding FLAG-UbVs or GFP (as control) respectively and 48 h pt these cells were infected with MERS-CoV at a multiplicity of infection of 0.01. Culture supernatants were collected 32 h post MERS-CoV infection and infectious progeny titers were determined by plaque assays. (**B**) Lentivirus transduced and MERS-CoV infected HuH-7 cells were 32 h post MERS-CoV infection lysed and expression of MERS-CoV nsp4, MERS-CoV ORF4B as well as expression of FLAG-UbVs or GFP was visualized via Western blotting.
(TIF)

S1 Table EC_50_ (Ub/UbV) and IC_50_ (UbV) values to cognate viral proteases. (PDF)

S2 Table Binding affinities of viral proteases and UbVs evaluated by Bio-Layer Interferometry (BLI). (PDF)
