# Supplementary material for: Potent and selective inhibition of pathogenic viruses by engineered ubiquitin variants
Source: PLoS Pathog. 2017 May 18;13(5):e1006372. doi: 10.1371/journal.ppat.1006372 (PMC5451084; doi:10.1371/journal.ppat.1006372)
Supplement: S1 Appendix — (DOCX) [file ppat.1006372.s001.docx]

**Supplemental Materials and Methods**

## ELISA assays to evaluate binding and specificity

Proteins under study were immobilized on 384-well MaxiSorp plates (Thermo Scientific 12665347) by adding 30 µL of 1 µM proteins for overnight incubation at 4 ºC. Phage and protein ELISA against immobilized proteins was performed as previously described [1, 2]. Binding of phage was detected using anti-M13-HRP antibody (GE Healthcare 27942101) and binding of FLAG-tagged UbVs was detected using anti-FLAG-HRP antibody (Sigma-Aldrich A8592). To measure the half maximal binding concentration (EC_50_) of UbVs binding to viral proteases, the concentration of UbVs or wild type Ub was varied from 0 to 4 µM (24 points, 1:2 dilution), while the concentration of target proteins immobilized on the plate remained at 1 µM. EC_50_ values were calculated using the GraphPad Prism software with the built-in equation formula (non-linear regression curve).

**Octet Bio-Layer Interferometry (BLI)**

Experiments were performed as previously described [2]. Concentrated analyte and ligand proteins were diluted into BLI reaction buffer (25 mM HEPES pH 7.0, 150 mM NaCl, 0.1 mg/ml bovine serum albumin and 0.01% tween 20). Experiments of BLI were carried out on Octet RED96 system (ForteBio) using anti-GST antibody biosensors for GST-tagged ligands and His-tagged analytes at 25˚C. 7-9 concentration points of analytes covering a wide titration range were applied for BLI experiments. Sensorgram raw data was processed and extracted by Octet Analysis 9.0. Binding constants *K_D_* were obtained by fitting the response wavelength shifts in the steady-state regions using single-site binding system (Eq. 1) shown below.

$R_{eq}=R_{max}\frac{[C]}{K_{D}+[C]}$

where *R_eq_* is the value of the response shift in the steady-state region in each sensorgram curve, *[C]* is the titrant concentration, *R_max_* is the maximal response in the steady-state region, *K_D_* is the binding constant for the single-site binding system. In both equations, *R_max_* and *K_D_* values are unknown and the Levenberg–Marquardt algorithm was used to perform iterative non-linear least squares curve fitting in Profit 6.2 (QuantumSoft) to obtain the fitted *R_max_* and *K_D_*.

**Deconjugation assays**

*Ub/ISG15-*amido-4-methylcoumarin *(AMC)*

Inhibition assays using Ub-AMC or ISG15-AMC (both Boston Biochem) as deconjugation substrates were performed as described before [1, 3]. Experiments were performed in assay buffer (50 mM HEPES, pH 7.5, 0.01% Tween 20, 1 mM dithiothreitol (DTT)) containing 1 μM Ub-AMC substrate, 10 nM viral DUBs (vDUBs) and 12 serial dilutions of UbVs. vDUBs and UbV were mixed in assay buffer as indicated and incubated at room temperature for 2 min prior to the addition of Ub-AMC. All serial dilutions were performed in 96-well plates and subsequently transferred to 384-well black plates (Thermo Scientific) for making measurements. Deconjugation activity was measured by monitoring the increase of AMC fluorescence emission at 460 nm (excitation at 360 nm) for 30 min using a BioTek Synergy2 plate reader (BioTek Instruments, Winooski, VT). IC_50_ values were calculated using the GraphPad Prism software with the built-in equation formula (non-linear regression curve).

*K48/K63 tetra-Ub*

Inhibition assays using Biotin Tetra-Ub/Ub4 WT Chains (K48- or K63-linked, Boston Biochem) as deubiquitination substrates were performed in assay buffer (50 mM HEPES, pH 7.5, 100 mM NaCl, 1 mM DTT) containing 1 μM substrate, 1 μM vDUB and 10 μM UbV as indicated. After incubation at 37°C for the indicated times, reactions were stopped by the addition of 10 mM EDTA and SDS-PAGE sample buffer and resolved using 4-20% gradient gels (Bio-Rad). The cleavage of K48/K63 tetra Ub chains was evaluated by Western blotting, probed with ExtrAvidin®-Peroxidase (Sigma).

**Protein expression and purification**

*Ubiquitin variants* *ME.2 and ME.4*

Plasmids named pET53-ME.2, and -ME.4 were transformed into CaCl_2_-competent *Escherichia coli* BL21 (DE3) Gold cells (Agilent) to allow for T7 polymerase-driven expression of N-terminally His_6_-tagged UbVs ME.2 and ME.4 respectively. Cells were grown at 37**°**C in the presence of 150 μg/mL ampicillin to an optical density (OD_600_) of 0.6 and then induced at 16**°**C by addition of Isopropyl β-D-1-thiogalactopyranoside (IPTG; final concentration 1 mM). After overnight incubation, cells were resuspended in lysis buffer (500 mM NaCl, 50 mM Tris pH 8.0) and lysed via French press. The cell lysate was clarified by centrifugation at 17,211 x g at 4**°**C for 30 min, then incubated with 2 mL Ni-NTA Superflow resin (Qiagen) at 4**°**C for 30 min, and poured into a gravity column. The column was washed with 50 mL of lysis buffer, followed by 50 mL of lysis buffer containing 50 mM imidazole. Protein was eluted in lysis buffer containing 250 mM imidazole. Following affinity purification, UbVs were further purified using a Superdex 75 size exclusion column (GE Healthcare), eluting in 20 mM Tris pH 8.5, 150 mM NaCl and 2 mM DTT.

*Ubiquitin variants* *CC.2 and CC.4*

Plasmids encoding UbVs CC.2 and CC.4, named pET53-CC.2 and -CC.4 respectively, were transformed into CaCl_2_-competent *E. coli* BL21 (DE3) Gold cells, and grown in Luria-Bertani media supplemented with 150 μg/ml Ampicillin at 37˚C with shaking to an OD_600_ of 0.8-1.5. Protein expression was induced by the addition of a final concentration of 1 mM IPTG for 19-21 hours at 28˚C. Cells were resuspended in lysis buffer (for CC.2: 50 mM Tris pH 7.2, 150 mM NaCl; for CC.4: 50 mM Tris pH 8.0, 150 mM NaCl) and lysed using either French Press or a freeze-thaw cycle that was performed three times. For the latter, cells were frozen at -80˚C after addition of 150 μl of 20 mg/ml lysozyme followed by incubation on ice for 20 min, and thawed at room temperature. Lysis was followed by addition of 100-300 μl of DNase I (10 mg/ml) while mixing on a magnetic stirrer. Lysates from French Press or the freeze-thaw procedure were clarified by centrifugation at 48,298 x g for 30-40 min. Supernatants were loaded onto a gravity column containing Ni-NTA Superflow resin (Qiagen) that had been pre-equilibrated with Buffer A (50 mM Tris pH 8.0, 150 mM NaCl). The column was washed with 2-3 column volumes (CV) of Buffer A supplemented with 20 mM imidazole and eluted in a step-wise manner with Buffer A containing 500 mM imidazole. Fractions of interest, as determined from SDS-PAGE, were pooled and dialyzed twice against Buffer A. The proteins were concentrated and loaded onto the Superdex 75 size exclusion column. CC.2 was eluted in 20 mM Tris pH 7.4, 150 mM NaCl and 1 mM DTT, and CC.4 was eluted in 50 mM Tris pH 8.0, 150 mM NaCl and 2 mM DTT.

*MERS-CoV PL^pro^*

The MERS-CoV PL^pro^ domain was expressed and purified as described previously [4]. Briefly, *E. coli* BL21 (DE3) Gold cells transformed with plasmid pE-SUMO PL^pro^ were grown to an OD_600_ ­of 0.6-0.8 at 37˚C in the presence of 35 μg/mL kanamycin. Protein expression was induced by the addition of a final concentration of 1 mM IPTG and overnight incubation at 16˚C. Cells were resuspended in lysis buffer (150 mM Tris, pH 8.5, 1 M NaCl, 2 mM DTT), lysed via French press and clarified via centrifugation at 17,211x g Clarified lysate was loaded onto a Ni-NTA gravity column and washed with lysis buffer, followed by lysis buffer supplemented with 25 mM imidazole and subsequent elution in lysis buffer supplemented with 250 mM imidazole. The SUMO-PL^pro^_­­_ fusion was cleaved overnight in 150 mM NaCl, 50 mM Tris, pH 8.0, 1 mM DTT in the presence of Ulp1 SUMO protease. Cleaved, tagless MERS-CoV PL^pro^ was subsequently passed through a second Ni-NTA column, and further purified on a Superdex 75 size exclusion column equilibrated in 20 mM Tris, pH 8.5, 150 mM NaCl, 2 mM DTT.

*CCHFV OTU_169_ and CCHFV OTU_185_*

Plasmids encoding the CCHFV OTU domain residues 1-169 (pGEX-*CCHFV OTU_169_)* and residues 1-185 (pET49b-*CCHFV OTU_185_*) fused with a GST tag and an HRV3c protease cleavage site were used for the expression and purification of the CCHFV OTU domain as described previously [5, 6]. Briefly, *E. coli* BL21-Gold (DE3) cells transformed with either of the plasmids were grown to an OD_600_ of 0.9-1.0 at 37˚C with shaking, and protein expression was induced with a final concentration of 1 mM IPTG at 30˚C for 19-21 hrs. Cells were resuspended in 25 ml of lysis buffer (50mM Tris-Cl pH 7.2, 200 mM NaCl, 5 mM EDTA, 5 mM DTT) and lysed via French Press. The lysate was clarified by centrifugation at 48,298 x g for 30-40 min. The supernatant was loaded onto GST-Bind resin (VWR) in a gravity column pre-equilibrated with the lysis buffer, and proteins were eluted in 50 mM Tris-Cl pH 7.2, 500 mM NaCl, 5 mM EDTA, 5 mM DTT and 15 mM reduced glutathione. The GST tag was cleaved by addition of GST-tagged HRV 3c protease during overnight dialysis against 50 mM Tris pH 7.4, 150 mM NaCl, 1 mM EDTA, 1 mM DTT. Cleaved CCHF-vOTU proteins were collected as flow-through by passing the digest through a recharged GST-Bind column, concentrated and loaded onto the Superdex-75 column (GE Healthcare). Purified proteins, eluted in 20 mM Tris pH 7.2, 150 mM NaCl and 1mM DTT, were then used for further analyses.

**Protein crystallization data collection**

*MERS-CoV PL_pro_-ME.2 and -ME.4 complexes*

Crystals were harvested by sweeping through a cryoprotectant solution containing 0.1M trisodium citrate pH 5.6, 22% (w/v) PEG 4000, 21% (v/v) 1,2-propanediol (PL^pro^-ME.4), or harvested directly from the crystallization solution (PL^pro^-ME.2) and flash-cooled in liquid nitrogen. X-ray diffraction data for PL^pro^-ME.4 crystals were collected using a Rigaku 007HF MicroFocus X-ray generator and R-AXIS IV++ detector. PL^pro^-ME.2 data collection was carried out at the Canadian Light Source on beamline 08B1-1. Diffraction data for the PL^pro^-ME.4 and –ME.2 crystals were integrated and scaled using XDS [7], followed by merging using Aimless within the CCP4 software suite. Initial phase estimates were determined via molecular replacement within phenix.phaser using the previously determined MERS-CoV PL^pro^ domain (PDB ID: 4RF0) and a polyAla Ub model (PDB ID: 4RF0) as independent search models [8]. Model building and refinement were performed using Coot and phenix.refine, respectively [9, 10].

*CCHFV OTU*_169_*-CC.2 and CCHFV OTU*_185_*-CC.4 complexes*

Single crystals of the CCHFV OTU_169_-CC.2 and CCHFV OTU_185_-CC.4 complexes were swept through cryoprotectant containing well solution supplemented with 20-30% ethylene glycol, and X-ray diffraction data were collected at the CLS on beamline 08ID-1 at 100K. X-ray diffraction data were indexed and scaled using XDS [7] and merged with Aimless [11]. Initial phases for the CCHFV OTU_169_-CC.2 and CCHFV OTU_185_-CC.4 complexes were determined by molecular replacement within phenix.phaser [8] using the previously reported CCHFV OTU domain bound to Ub (PDB 3PT2) [5] as a search model. The structures of CC.2 and CC.4 bound to CCHFV OTU were subsequently modelled and refined into the MR maps using Coot [9] and phenix.refine, respectively [10].

## Plasmids used for cell culture work

The following plasmids were described elsewhere or provided by others: pcDNA3.1-MERS-CoV-PL^pro^ WT and active site mutant C1592A [12], pCAGGS-HA-nsp3C-4-V5 [12], pcDNA-eGFP [13], pCAGGS- MAVS (provided by N. Frias-Staheli), pLuc-IFN-β [14] and pRL-TK (Promega). pcDNA3-HA-Ub was generated by cloning PCR-amplified Ub (using pCMV-FLAG-Ub [15] as a template) in pcDNA3.1(-) (Invitrogen) in frame with an N-terminal HA tag. Codon optimized SARS-CoV-PL^pro^ (amino acids 1541-1855 of the SARS-CoV pp1a/pp1ab (NCBI ID: AY291315.1)) with removed potential splice sites and polyadenylation signals (IDT) was cloned into pcDNA3.1(-) in frame with a C-terminal V5-tag generating pcDNA3.1(-)-SARS-CoV-PL^pro^-V5. pcDNA3.1(-)-SARS-CoV-PL^pro^-V5 was used as template for site-directed mutagenesis using the QuikChange^TM^ strategy (Stratagene using Accuzyme DNA polymerase from Bioline) to mutate the active site cysteine to alanine (C1651A).

In the UbV-containing pDONR-221 vectors (Thermo Fisher Scientific) a methionine was introduced before the already present N-terminal FLAG tag using the QuikChange^TM^ strategy. In pDONR-221-FLAG-Ub substitutions G75A+G76A were introduced in Ub to generate pDONR-221-FLAG-Ub.AA. FLAG-UbVs were cloned in destination vectors pcDNA3.1-DEST or pLenti6.3/TO/V5-DEST (Thermo Fisher Scientific) using the Gateway technology (Thermo Fisher Scientific). Primer sequences are available upon request.

## Cell culture and antibodies

HEK293T were grown in Dulbecco’s modified Eagle’s medium (DMEM) supplemented with 10% fetal calf serum (FCS; Bodinco BV), 100 units/ml penicillin, 100 units/ml streptomycin and 2 mM L-glutamine. Vero cells (Erasmus Medical Center) and MRC5 cells (ATCC CCL-171) were cultured in Eagle’s minimum essential medium (EMEM) with 8% FCS, 100 units/ml penicillin and streptomycin, 2 mM L-glutamine and non-essential amino acids (PAA). HuH-7 cells were maintained in DMEM containing 8% FCS, antibiotics and non-essential amino acids. DMEM, EMEM and supplements were obtained from Lonza.

Proteins on Western blot were visualized using the following primary antibodies: mouse anti-FLAG (F3165; Sigma-Aldrich), mouse anti-V5 (37-7500; Invitrogen), mouse anti-HA (ab18181; Abcam), rabbit anti-GFP [13], rabbit anti-SARS-CoV nsp4 [16], rabbit anti-MERS-CoV p4b [17] and mouse anti-actin (A5316; Sigma-Aldrich). Primary antibodies were detected with horseradish peroxidase-conjugated secondary antibodies (P0447 and P0217; Dako). In an indirect immunofluorescence assay primary antibody mouse anti-FLAG (F3165; Sigma-Aldrich) and secondary antibody Alexa488-conjugated goat anti-mouse immunoglobulin G (IgG) antibody (A-11001; Thermo Fisher Scientific) were used.

## Protease activity assays in cell culture

Protease activity assays as described in Bailey-Elkin et al. [12] were performed with slight modifications. Briefly, to assess the DUB activity of MERS-CoV PL^pro^ in the presence of UbVs, HEK293T cells were co-transfected using the calcium phosphate transfection method with plasmids encoding HA-tagged Ub (0.25 μg), MERS-CoV-PL^pro^-V5 (0.2 μg), FLAG-tagged UbVs (0.5; 0.75 or 1 μg, as indicated) and GFP (0.25 μg). In order to establish whether the MERS-CoV-directed UbVs target SARS-CoV PL^pro^, DUB assays were also performed with SARS-CoV-PL^pro^-V5 (0.2 μg) instead of MERS-CoV-PL^pro^-V5. The *in trans* cleavage activity of MERS-CoV PL^pro^ in the presence of UbVs was determined by co-expressing HA-nsp3C-4-V5 (0.2 μg), MERS-CoV-PL^pro^-V5 (0.15 μg), FLAG-tagged UbVs (0.5; 0.75 or 1 μg) and GFP (0.25 μg) in HEK293T cells. Empty pcDNA vector was added to supplement to a total of 2 μg of plasmid DNA transfected per well of a 12-wells cluster. At 18 h post transfection, cells were lysed in 2x Laemmli sample buffer (LSB) containing 25 mM N-ethylmaleimide (NEM; Sigma). Proteins were separated in an SDS-PAGE gel, blotted onto Hybond-P (0,45 μm pore size, GE-Heathcare) and visualized after antibody incubation steps using Pierce ECL 2 Western blotting substrate (Thermo Fisher Scientific). To visualize FLAG-tagged UbVs the proteins separated in an SDS-page gel were blotted onto 0,2 μm PVDF membranes (GE-Heathcare). The membranes were blocked with dried milk powder in PBS containing 0,05% Tween-20 followed by antibody incubation steps.

## Luciferase-based IFN-β reporter assay

In a luciferase-based IFN-β reporter assay the effect of the UbVs on the suppression of IFN-β promoter activity by viral PL^pro^s was investigated. HEK293T cells grown to 80% confluency in a 24-wells plate were co-transfected with a combination of plasmids encoding the firefly luciferase reporter gene under control of the IFN-β promoter (25 ng), *Renilla* luciferase (5 ng), innate immune inducer mitochondrial antiviral signalling protein (MAVS; 25 ng), MERS-CoV PL^pro^-V5 (250 ng) and FLAG-tagged UbVs (250, 500, 750 ng). Alternatively, SARS-CoV PL^pro^-V5 (100 ng) and FLAG-tagged UbVs (750 ng) were co-expressed with firefly luciferase under control of the IFN-β promoter, *Renilla* luciferase and MAVS. Total amounts of transfected DNA were equalized by the addition of empty pcDNA vector. Both firefly and *Renilla* luciferase activities were measured (Mithras LB 940 multimode reader; Berthold Technologies) using the Dual-Luciferase reporter assay system (Promega) 16 h post transfection. After normalizing the firefly luciferase activity to *Renilla* luciferase activity, an unpaired two-tailed Student’s t test was performed and p values <0.05 were considered statistically significant. Firefly and *Renilla* luciferase activities were measured in triplicate and assays were repeated independently at least three times.

**References**

1. Ernst A, Avvakumov G, Tong J, Fan Y, Zhao Y, Alberts P, et al. A strategy for modulation of enzymes in the ubiquitin system. Science. 2013;339(6119):590-5. Epub 2013/01/05. doi: 10.1126/science.1230161. PubMed PMID: 23287719.

2. Zhang W, Wu KP, Sartori MA, Kamadurai HB, Ordureau A, Jiang C, et al. System-Wide Modulation of HECT E3 Ligases with Selective Ubiquitin Variant Probes. Molecular cell. 2016;62(1):121-36. doi: 10.1016/j.molcel.2016.02.005. PubMed PMID: 26949039.

3. Leung I, Dekel A, Shifman JM, Sidhu SS. Saturation scanning of ubiquitin variants reveals a common hot spot for binding to USP2 and USP21. Proceedings of the National Academy of Sciences of the United States of America. 2016. doi: 10.1073/pnas.1524648113. PubMed PMID: 27436899.

4. Bailey-Elkin BA, van Kasteren PB, Snijder EJ, Kikkert M, Mark BL. Viral OTU deubiquitinases: a structural and functional comparison. PLoS Pathog. 2014;10(3):e1003894. doi: 10.1371/journal.ppat.1003894. PubMed PMID: 24676359; PubMed Central PMCID: PMCPMC3968130.

5. James TW, Frias-Staheli N, Bacik JP, Levingston Macleod JM, Khajehpour M, Garcia-Sastre A, et al. Structural basis for the removal of ubiquitin and interferon-stimulated gene 15 by a viral ovarian tumor domain-containing protease. Proceedings of the National Academy of Sciences of the United States of America. 2011;108(6):2222-7. doi: 10.1073/pnas.1013388108. PubMed PMID: 21245344; PubMed Central PMCID: PMCPMC3038750.

6. Frias-Staheli N, Giannakopoulos NV, Kikkert M, Taylor SL, Bridgen A, Paragas J, et al. Ovarian tumor domain-containing viral proteases evade ubiquitin- and ISG15-dependent innate immune responses. Cell Host Microbe. 2007;2(6):404-16. doi: 10.1016/j.chom.2007.09.014. PubMed PMID: 18078692; PubMed Central PMCID: PMCPMC2184509.

7. Kabsch W. Xds. Acta Crystallogr D Biol Crystallogr. 2010;66(Pt 2):125-32. doi: 10.1107/S0907444909047337. PubMed PMID: 20124692; PubMed Central PMCID: PMCPMC2815665.

8. McCoy AJ, Grosse-Kunstleve RW, Adams PD, Winn MD, Storoni LC, Read RJ. Phaser crystallographic software. J Appl Crystallogr. 2007;40(Pt 4):658-74. doi: 10.1107/S0021889807021206. PubMed PMID: 19461840; PubMed Central PMCID: PMCPMC2483472.

9. Emsley P, Lohkamp B, Scott WG, Cowtan K. Features and development of Coot. Acta Crystallogr D Biol Crystallogr. 2010;66(Pt 4):486-501. doi: 10.1107/S0907444910007493. PubMed PMID: 20383002; PubMed Central PMCID: PMCPMC2852313.

10. Afonine PV, Grosse-Kunstleve RW, Echols N, Headd JJ, Moriarty NW, Mustyakimov M, et al. Towards automated crystallographic structure refinement with phenix.refine. Acta Crystallogr D Biol Crystallogr. 2012;68(Pt 4):352-67. doi: 10.1107/S0907444912001308. PubMed PMID: 22505256; PubMed Central PMCID: PMCPMC3322595.

11. Evans PR. An introduction to data reduction: space-group determination, scaling and intensity statistics. Acta Crystallogr D Biol Crystallogr. 2011;67(Pt 4):282-92. doi: 10.1107/S090744491003982X. PubMed PMID: 21460446; PubMed Central PMCID: PMCPMC3069743.

12. Bailey-Elkin BA, Knaap RC, Johnson GG, Dalebout TJ, Ninaber DK, van Kasteren PB, et al. Crystal structure of the Middle East respiratory syndrome coronavirus (MERS-CoV) papain-like protease bound to ubiquitin facilitates targeted disruption of deubiquitinating activity to demonstrate its role in innate immune suppression. The Journal of biological chemistry. 2014;289(50):34667-82. doi: 10.1074/jbc.M114.609644. PubMed PMID: 25320088; PubMed Central PMCID: PMCPMC4263872.

13. van Kasteren PB, Beugeling C, Ninaber DK, Frias-Staheli N, van Boheemen S, Garcia-Sastre A, et al. Arterivirus and nairovirus ovarian tumor domain-containing Deubiquitinases target activated RIG-I to control innate immune signaling. J Virol. 2012;86(2):773-85. doi: 10.1128/JVI.06277-11. PubMed PMID: 22072774; PubMed Central PMCID: PMCPMC3255818.

14. Fitzgerald KA, McWhirter SM, Faia KL, Rowe DC, Latz E, Golenbock DT, et al. IKKepsilon and TBK1 are essential components of the IRF3 signaling pathway. Nat Immunol. 2003;4(5):491-6. doi: 10.1038/ni921. PubMed PMID: 12692549.

15. Gack MU, Albrecht RA, Urano T, Inn KS, Huang IC, Carnero E, et al. Influenza A virus NS1 targets the ubiquitin ligase TRIM25 to evade recognition by the host viral RNA sensor RIG-I. Cell host & microbe. 2009;5(5):439-49. doi: 10.1016/j.chom.2009.04.006. PubMed PMID: 19454348; PubMed Central PMCID: PMCPMC2737813.

16. van Hemert MJ, van den Worm SH, Knoops K, Mommaas AM, Gorbalenya AE, Snijder EJ. SARS-coronavirus replication/transcription complexes are membrane-protected and need a host factor for activity in vitro. PLoS pathogens. 2008;4(5):e1000054. doi: 10.1371/journal.ppat.1000054. PubMed PMID: 18451981; PubMed Central PMCID: PMC2322833.

17. Matthews KL, Coleman CM, van der Meer Y, Snijder EJ, Frieman MB. The ORF4b-encoded accessory proteins of Middle East respiratory syndrome coronavirus and two related bat coronaviruses localize to the nucleus and inhibit innate immune signalling. The Journal of general virology. 2014;95(Pt 4):874-82. doi: 10.1099/vir.0.062059-0. PubMed PMID: 24443473; PubMed Central PMCID: PMC3973478.
