# Supplementary figures and images for: Potent and selective inhibition of pathogenic viruses by engineered ubiquitin variants
Source: PLoS Pathog. 2017 May 18;13(5):e1006372. doi: 10.1371/journal.ppat.1006372 (PMC5451084; doi:10.1371/journal.ppat.1006372)

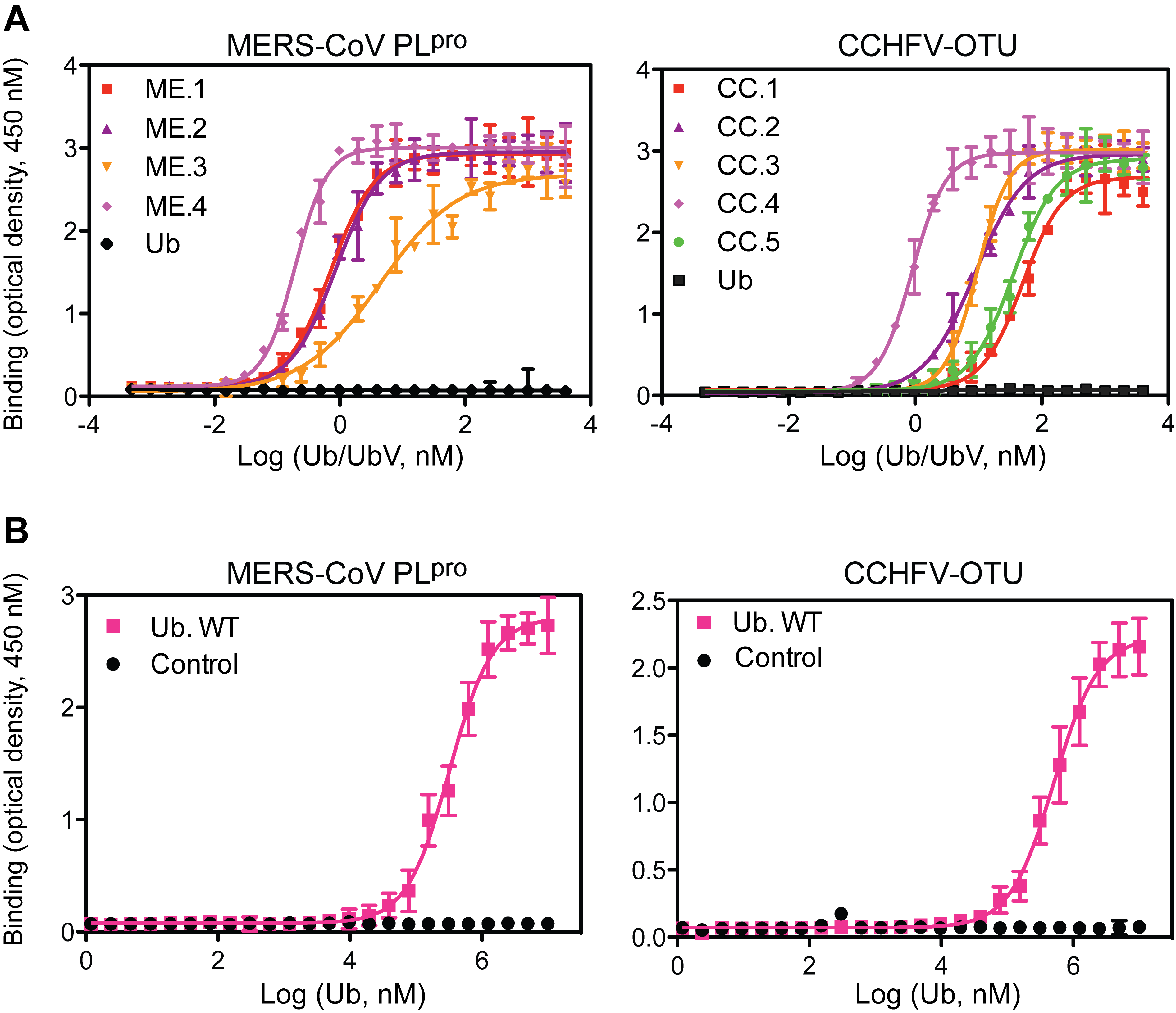

Supplement: S1 Fig — (A) Binding curves of UbVs to the cognate viral proteases (left panel: MERS-CoV PLpro; right panel: CCHFV OTU), measured by ELISA. The half maximal binding concentrations (EC50) of UbVs to indicated vDUBs were determined by established methods [26] and are listed in S1 Table. Viral proteases (1 μM) were immobilized in microtiter plates. Serial dilutions of FLAG-tagged UbV or Ub (up to 4 μM, 24 points) were added and incubated for 20 min at room temperature. Wells were washed and bound UbV/Ub was detected by anti-FLAG-HRP conjugate antibody and colorimetric development of TMB peroxidase substrate. The absorbance at 450 nm (y-axis) was plotted against Log (UbV/Ub concentration, nM) (x-axis). Data were presented as the mean ± SD (N = 3). (B) Binding curves of wild type Ub (Ub.wt) to MERS-CoV PLpro (left) and CCHFV OTU (right). Experiments were performed as described in (A) except the concentration of Ub was increased (up to 10 mM, 24 serial dilutions). Data were presented as the mean ± SD (N = 3). (TIF) [file ppat.1006372.s002.tif]

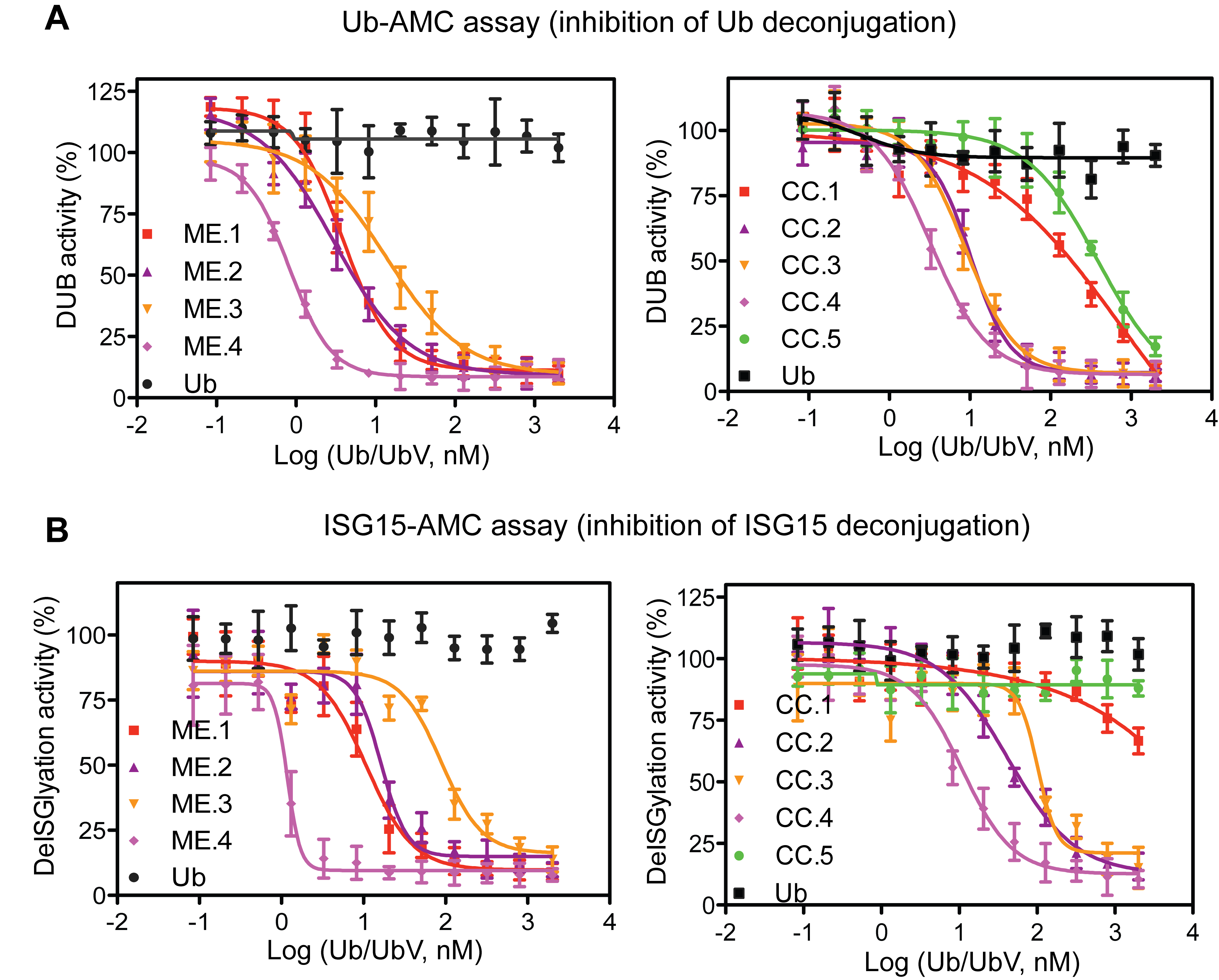

Supplement: S2 Fig — (A-B) Inhibition of MERS-CoV PLpro (left) or CCHFV OTU (right) by the cognate UbVs shown as dose-response curves using Ub-AMC (A) or ISG15-AMC (B) as a substrate. The IC50 values were determined as the concentrations of UbVs that reduced deubiquitination or deISGylation activity by 50% (S1 Table). The wt Ub data obtained in the deISGylation assay can not be fitted by GraphPad Prism so no lines were shown. (TIF) [file ppat.1006372.s003.tif]

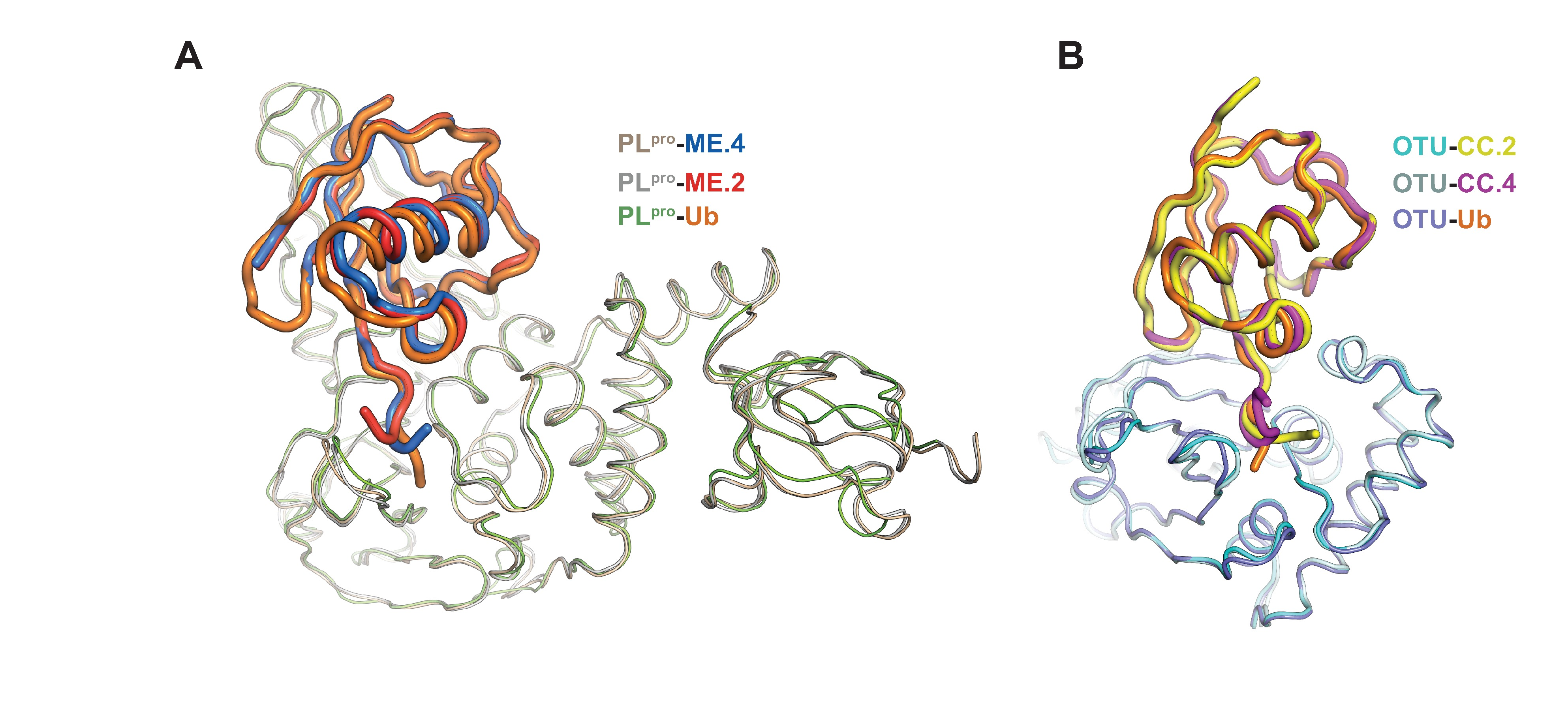

Supplement: S3 Fig — (A) Superposition of the MERS-CoV PLpro-Ub.wt, -ME.2 and -ME.4 complexes. PLpro is displayed as ribbons, and coloured in chartreuse, gray and wheat in the PLpro-Ub.wt, -ME.2 and -ME.4 structures, respectively. The Ub and UbV structures are displayed as tubes, and coloured in orange, red and marine in the PLpro-Ub.wt, -ME.2 and -ME.4 structures, respectively. (B) Superposition of the CCHFV OTU-Ub.wt, -CC.2 and CC.4 complexes. CCHFV OTU is displayed as ribbons, and coloured in slate, cyan and pale cyan in the CCHFV OTU-Ub.wt, -CC.2 and -CC.4 structures, respectively. The Ub and UbV structures are displayed as tubes, and coloured in orange, yellow and magenta in the CCHFV OTU-Ub.wt, -CC.2 and -CC.4 structures, respectively. Structures were aligned within PyMOL [61]. (TIF) [file ppat.1006372.s004.tif]

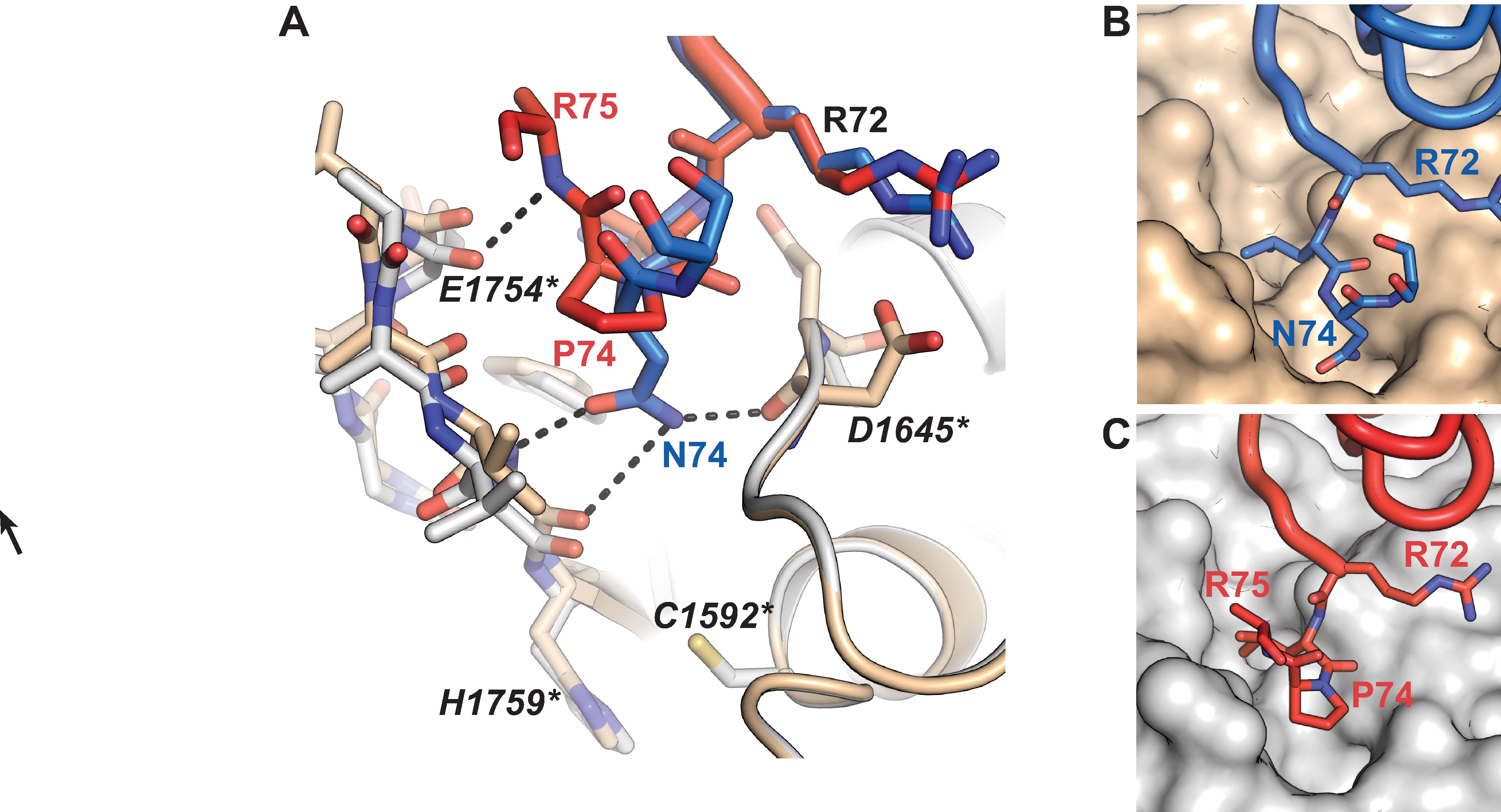

Supplement: S4 Fig — (A) Superposition of the C-terminal regions of the MERS-CoV PLpro-ME.2 and–ME.4 structures. PLpro is coloured in gray and wheat in the MERS-CoV PLpro-ME.2 and–ME.4 structures, and ME.2 and ME.4 are coloured in red and marine, respectively. PLpro active site residues His1759 and Cys1592 are shown as sticks, along with additional PLpro, ME.2 and ME.4 residues involved in binding. (B) Close up of the C-terminus of ME.4 in the MERS-CoV PLpro-ME.4 complex, with PLpro depicted in a surface representation. (C) Close up of the C-terminus of ME.2 in the MERS-CoV PLpro-ME.2 complex, with PLpro depicted in a surface representation. (TIF) [file ppat.1006372.s005.tif]

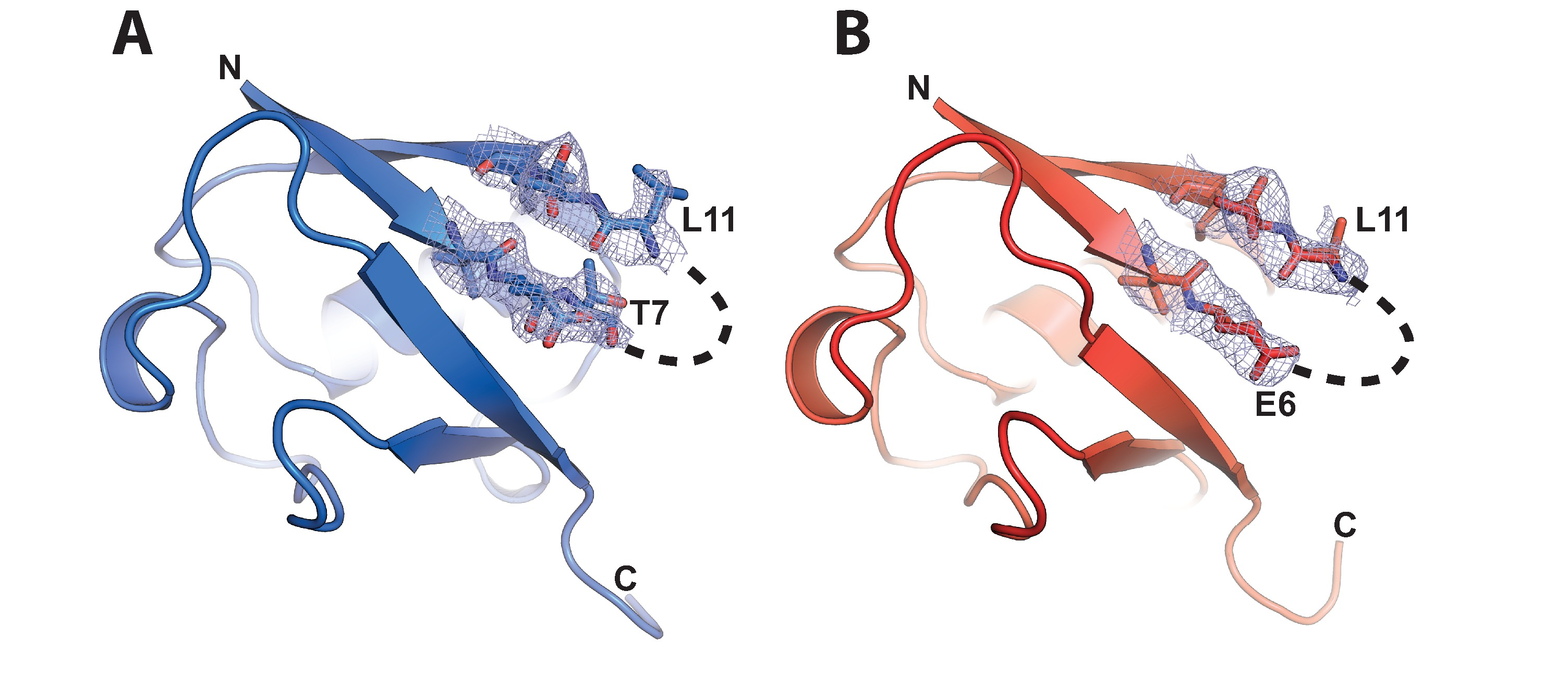

Supplement: S5 Fig — (A) Cartoon representation of ME.4 (marine). Dashed line indicates missing residues 8–10 which were not resolved in the electron density maps. A 2Fo-Fc electron density map is displayed as blue mesh and contoured at 1.0 RMSD. (B) Cartoon representation of ME.2 (red). Dashed line indicates missing residues 7–10. Figure generated with PyMOL [61]. (TIF) [file ppat.1006372.s006.tif]

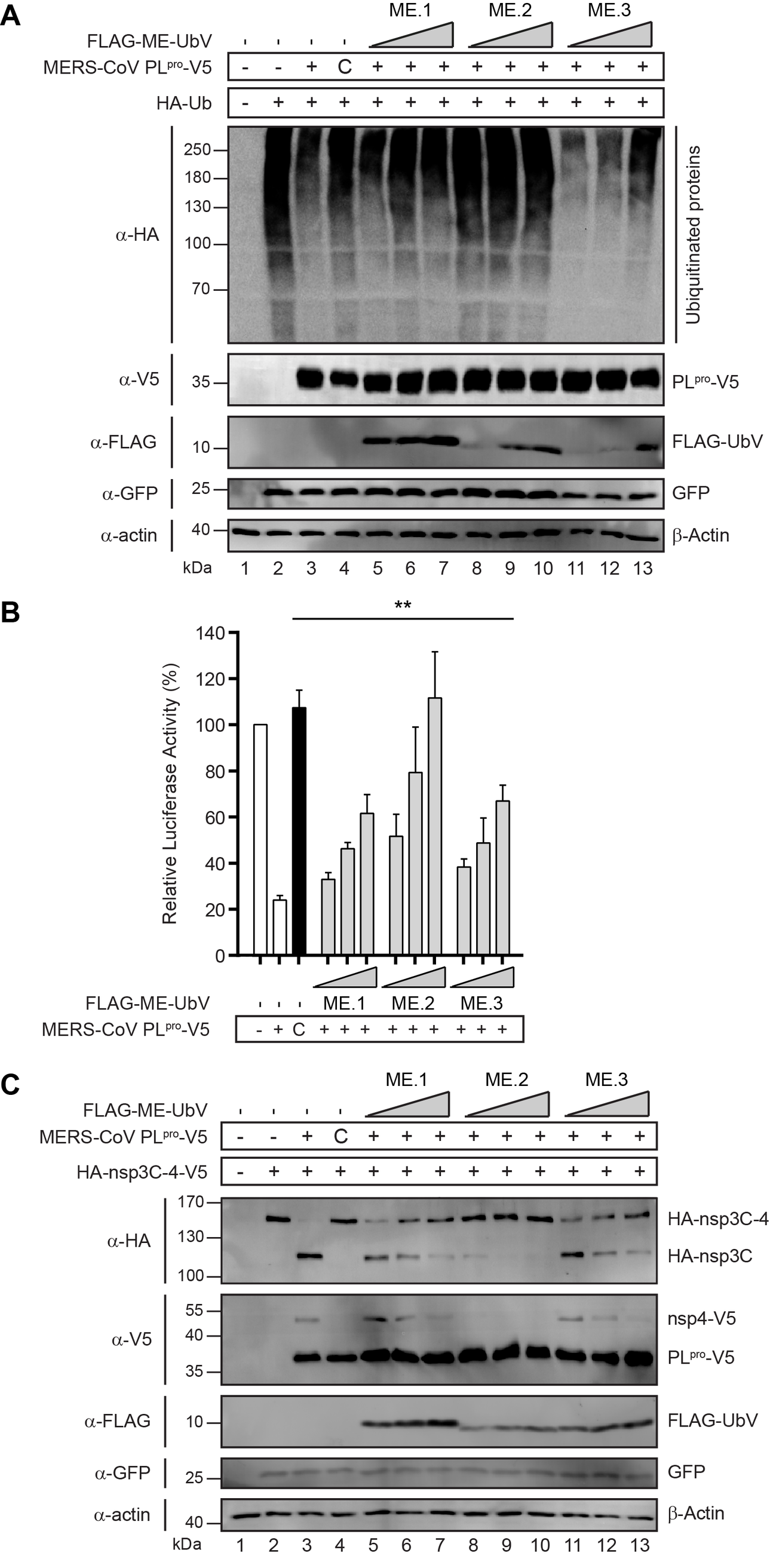

Supplement: S6 Fig — (A) Inhibition of MERS-CoV PLpro DUB activity by ME.1, ME.2 and ME.3 was determined by expressing HA-Ub, MERS-CoV PLpro-V5 (wild type or the active site mutant C1592A designated as C), FLAG-ME-UbV (500, 750 or 1000 ng of the appropriate plasmid) and GFP (as a transfection control) in HEK293T cells. After obtaining protein lysates the expressed proteins were separated on a SDS-PAGE gel, blotted and visualized after antibody incubations. (B) Suppression of the IFN-β promoter activity by MERS-CoV PLpro in the presence of UbVs was assessed by transfecting plasmids encoding firefly luciferase reporter gene under control of the IFN-β promoter, Renilla luciferase, MAVS, MERS-CoV PLpro-V5 (wild type or the active site mutant C) and FLAG-tagged UbVs (250, 500 or 750 ng). Firefly and Renilla luciferase activities were measured 16 h post transfection and significance relative to wild-type without expression of a UbV was calculated using an unpaired two-tailed Student’s t test. Significant values were indicated: ** p < 0.01. Bars represent mean and error bars represent S.D (N = 3). (C) Proteolytic cleavage capability of MERS-CoV PLpro was assessed in the presence of the UbVs. N-terminally HA-tagged and C-terminally V5-tagged nsp3C-4 (a polyprotein fragment excluding PLpro) was co-expressed with MERS-CoV PLpro-V5 (wild type or the active site mutant C), FLAG-ME-UbV (at increasing concentrations) and GFP (as a transfection control). Cells were lysed 18 h post-transfection and expressed proteins were analyzed by Western blotting. (TIF) [file ppat.1006372.s007.tif]

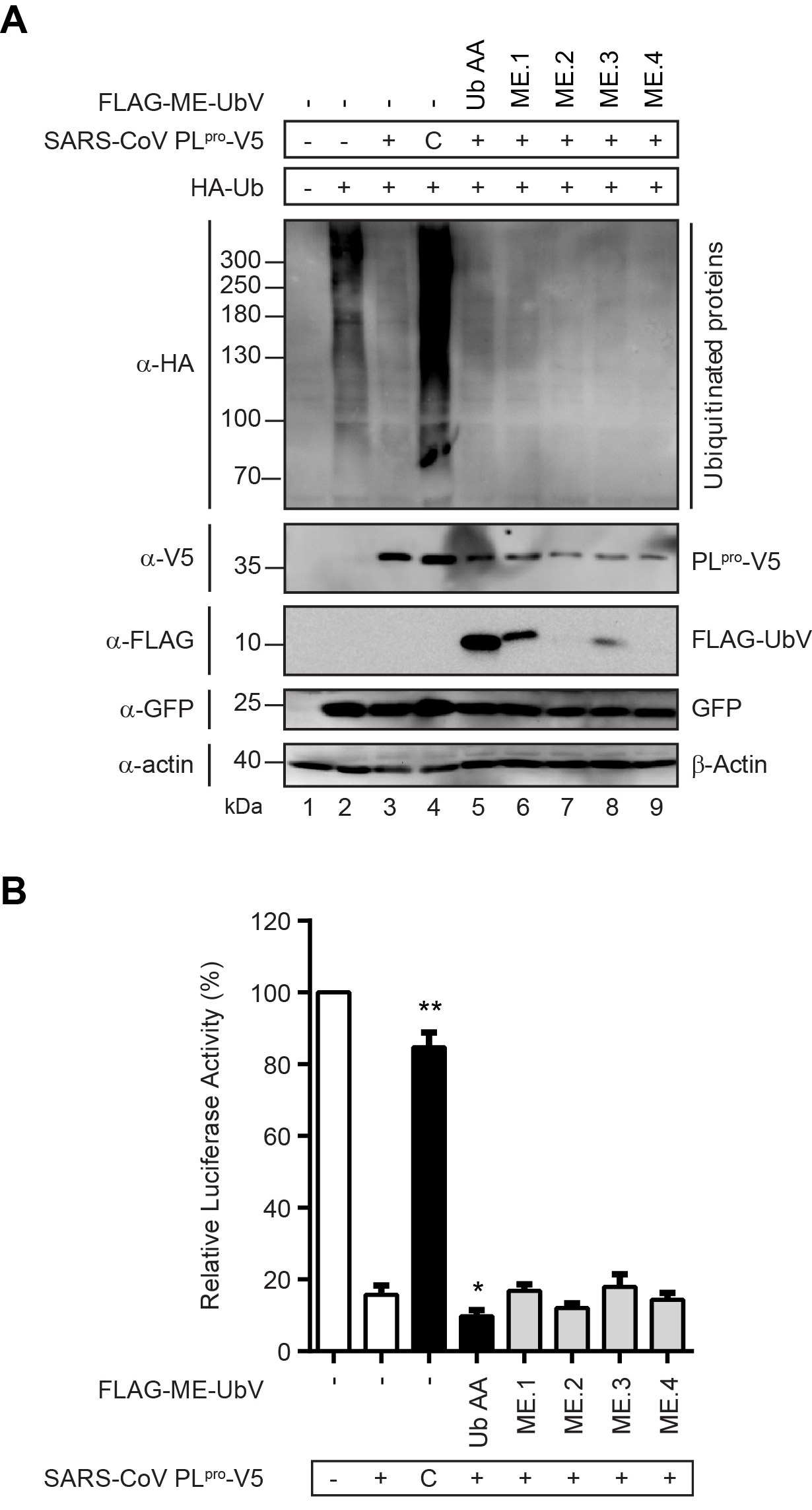

Supplement: S7 Fig — (A) SARS-CoV PLpro’s DUB activity in the presence of UbVs was determined by co-transfecting HEK293T cells with plasmids encoding HA-Ub, SARS-CoV PLpro-V5 (wild type or the active site mutant C1651A designated as C), FLAG-ME-UbV (1000 ng) and GFP (as a transfection control). 18 h post-transfection cells were lysed and deconjugation of HA-tagged Ub by SARS-CoV PLpro was visualized via Western blotting. (B) HEK293T cells were transfected with plasmids encoding firefly luciferase reporter gene under control of the IFN-β promoter, Renilla luciferase, MAVS, SARS-CoV PLpro-V5 (wild type or the active site mutant C; 100 ng) and FLAG-tagged UbVs (750 ng). Cells were lysed 16 h post-transfection and both firefly and Renilla luciferase activities were measured. Significance relative to wild-type without expression of a UbV was measured using an unpaired two-tailed Student’s t test; significant values were indicated: ** p < 0.01. Bars represent mean and error bars represent S.D (N = 3). (TIF) [file ppat.1006372.s008.tif]

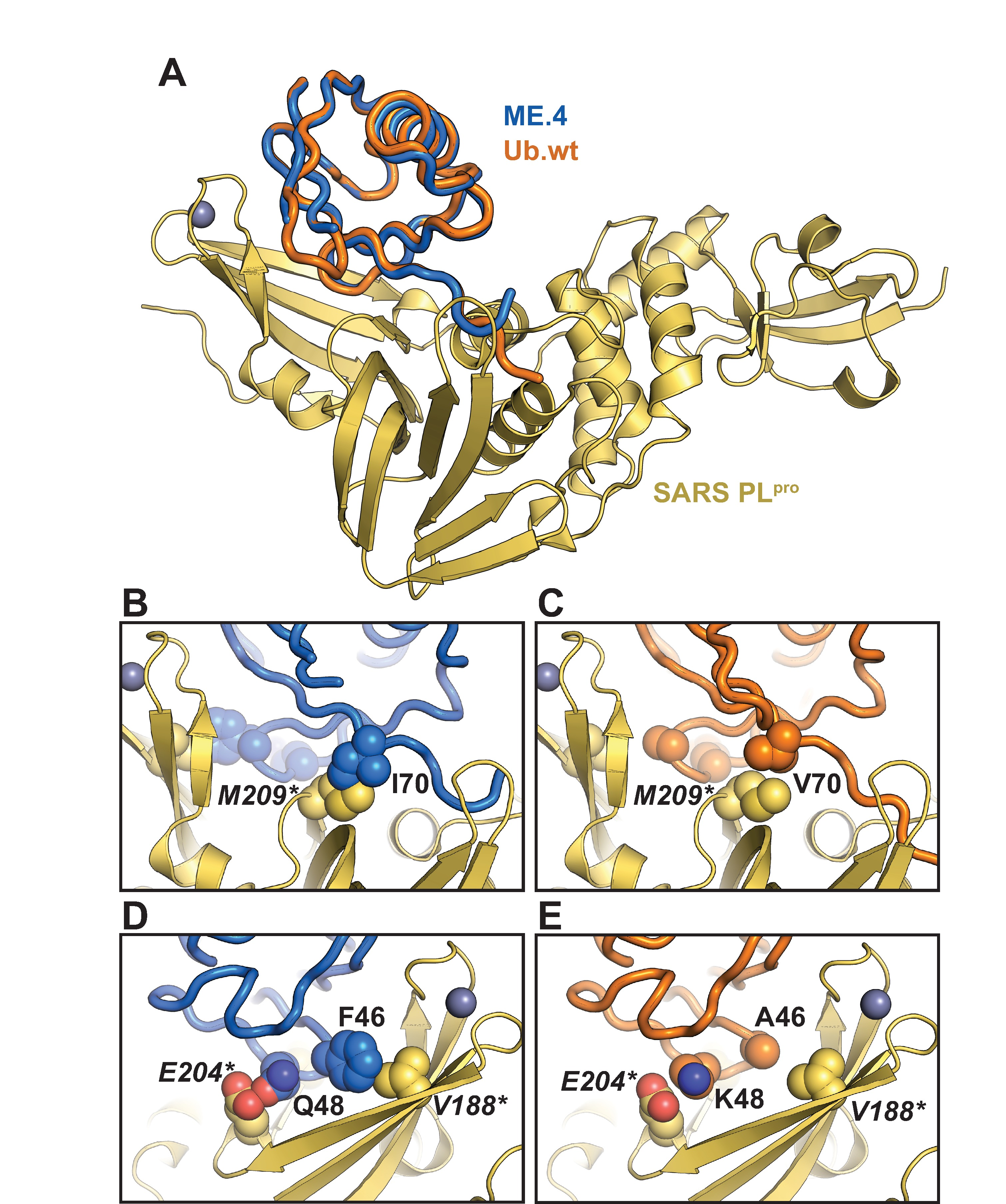

Supplement: S8 Fig — (A) The SARS-CoV PLpro domain is shown as a cartoon representation (yellow-orange). ME.4 and Ub.wt are shown in tubes (marine and orange, respectively). The ME.4 structure determined herein was superposed over Ub bound to the SARS-CoV PLpro domain (4M0W [62]) (B) Close-up of residue clashes occurring between SARS-CoV PLpro and ME.4. Residues are shown as spheres, with SARS-CoV PLpro residues indicated with asterisks and in italics. SARS-CoV PLpro residue M209 clashed with ME.4 residue I70, compared with Ub residue V70 (C). (D) SARS-CoV PLpro residues E204 and V188 clash with ME.4 residues Q48 and V188, respectively, compared to Ub.wt residues K48 and A46 (E). Figure generated in PyMOL [61]. (TIF) [file ppat.1006372.s009.tif]

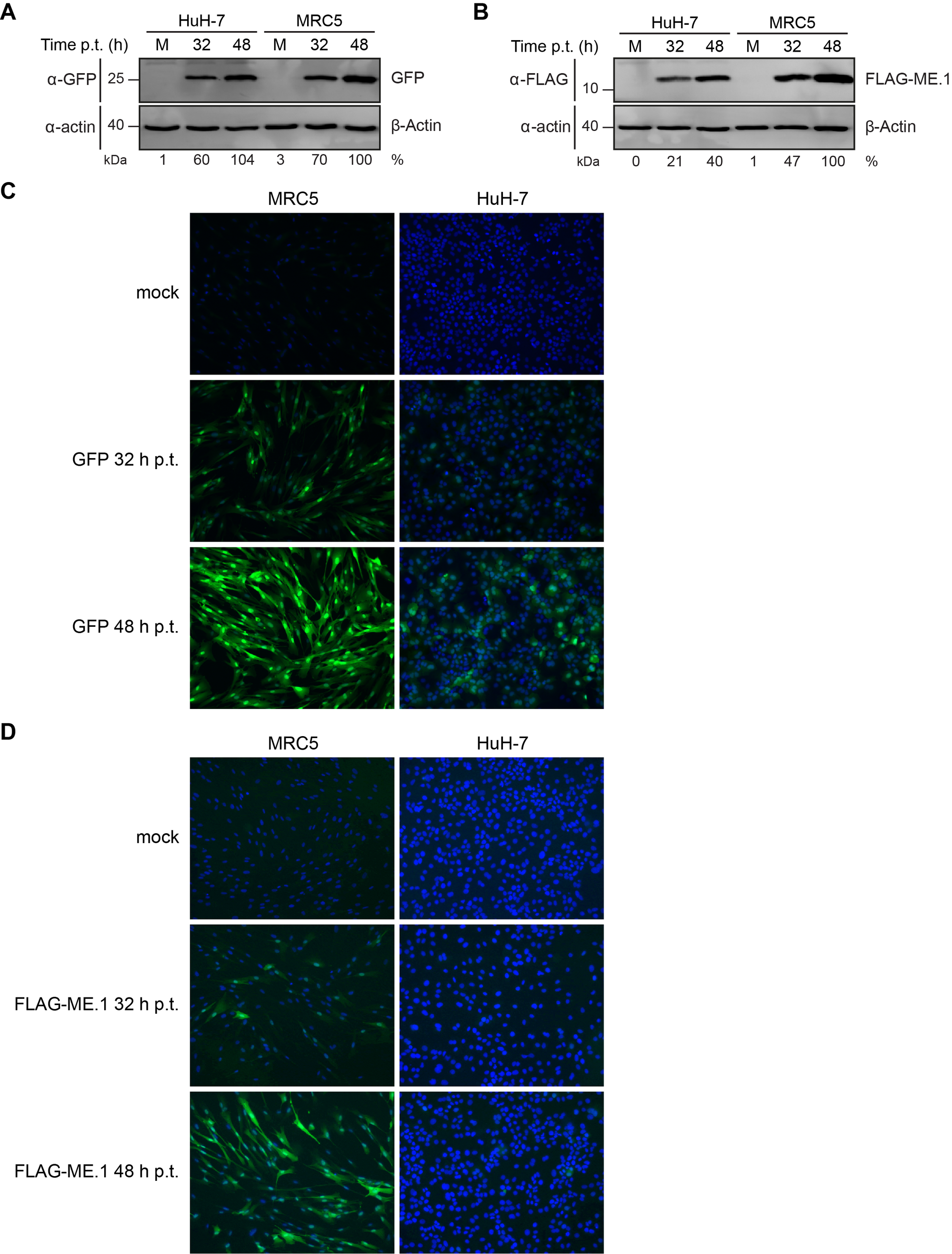

Supplement: S9 Fig — (A, B) Western blot analysis of transduced MRC5 and HuH-7 cells with lentiviruses encoding GFP (A) or FLAG-ME.1 (B) both 32 h and 48 h pt. As a control cells were mock transduced (designated as M). Relative expression of GFP and FLAG-ME.1 was quantified and normalized to actin and expression levels in MRC5 cells 48 h pt were set at 100%. (C) GFP transduced MRC5 and HuH-7 cells were fixed 32 h or 48 h pt and nuclear DNA was stained using Hoechst. Images were taken using fixed exposure times for both the GFP and Hoechst signal. (D) Immunofluorescence assay of FLAG-ME.1-transduced MRC5 and HuH-7 cells that were fixed 32 h or 48 h pt. Cells were labelled with a mouse monoclonal antibody recognizing FLAG followed by labelling with a secondary Alexa488-conjugated goat anti-mouse antibody. Exposure times were kept the same for each image. (TIF) [file ppat.1006372.s010.tif]

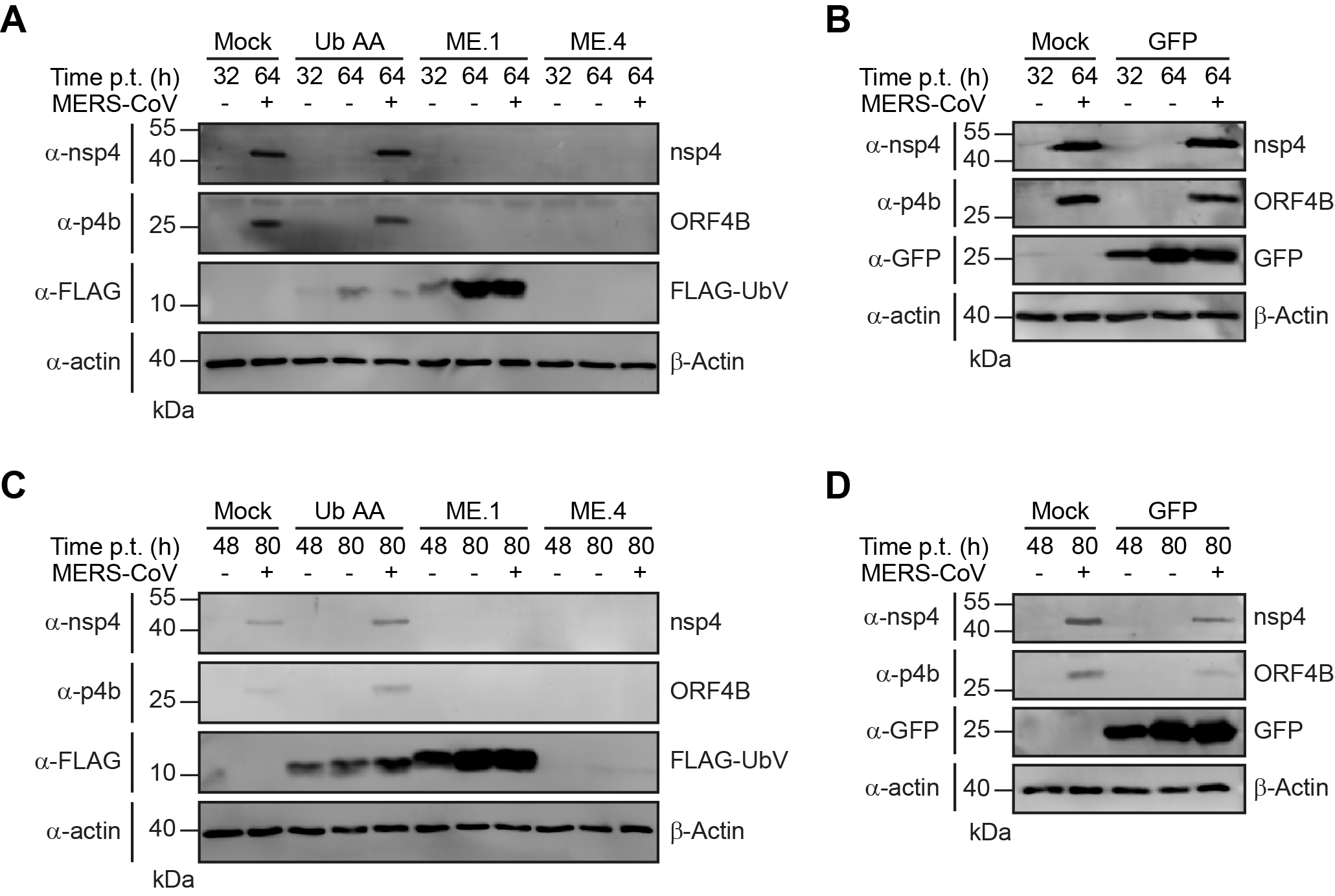

Supplement: S10 Fig — Upon collection of supernatants after MERS-CoV infection of transduced MRC5 cells protein lysates were obtained. Expression of two viral proteins was analyzed by Western blotting, MERS-CoV nsp4 (using cross-reacting SARS-CoV nsp4 antiserum), and MERS-CoV ORF4B. Lentivirus-induced expression of FLAG-UbVs or GFP was confirmed and actin was used as a loading control. Representative Western blots are shown for transduced MRC5 cells that were infected with MERS-CoV at a multiplicity of infection of 0.01 either 32 h pt (A, B) or 48 h pt (C, D). (TIF) [file ppat.1006372.s011.tif]

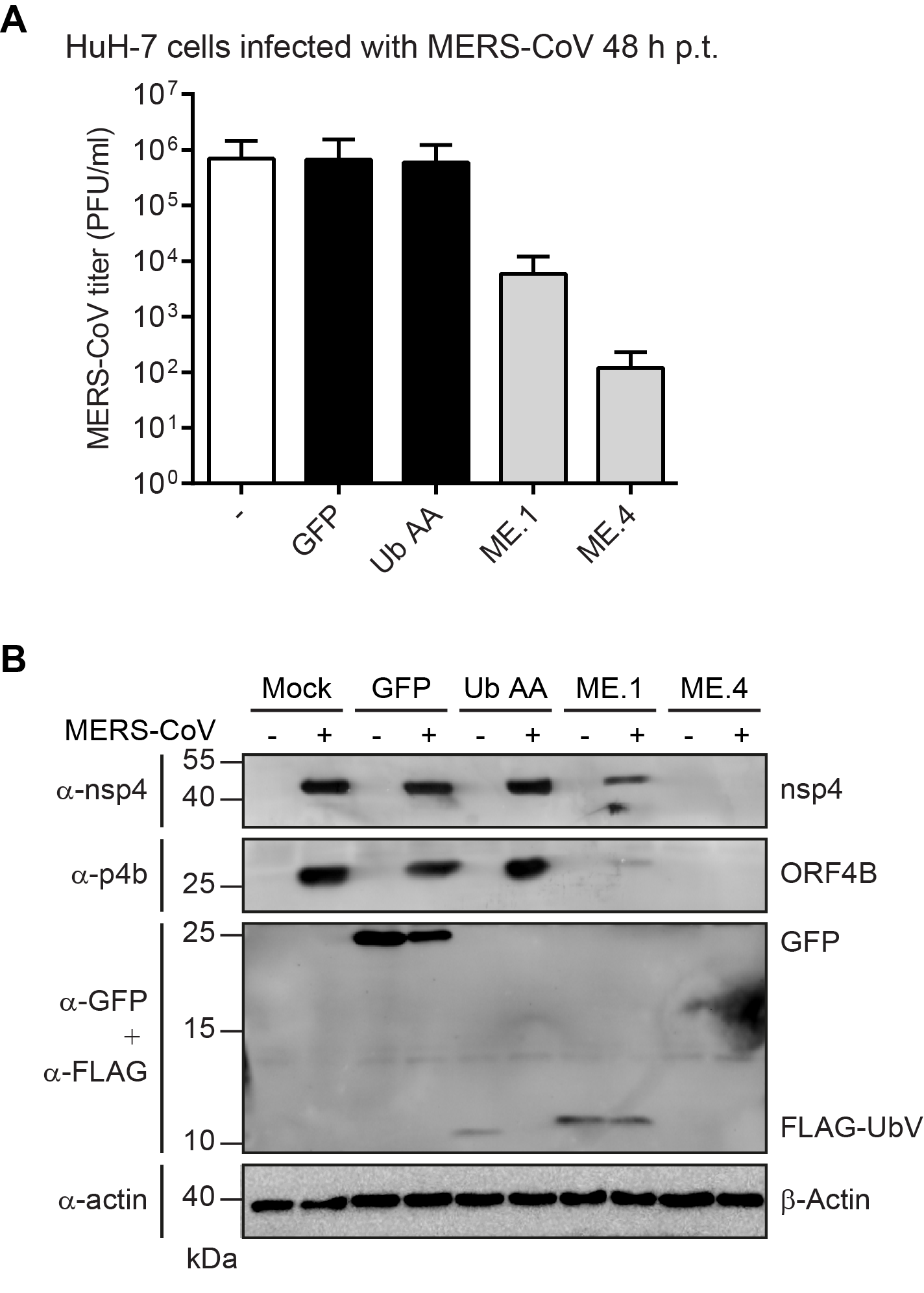

Supplement: S11 Fig — (A) HuH-7 cells were transduced with lentiviruses encoding FLAG-UbVs or GFP (as control) respectively and 48 h pt these cells were infected with MERS-CoV at a multiplicity of infection of 0.01. Culture supernatants were collected 32 h post MERS-CoV infection and infectious progeny titers were determined by plaque assays. (B) Lentivirus transduced and MERS-CoV infected HuH-7 cells were 32 h post MERS-CoV infection lysed and expression of MERS-CoV nsp4, MERS-CoV ORF4B as well as expression of FLAG-UbVs or GFP was visualized via Western blotting. (TIF) [file ppat.1006372.s012.tif]
