# Supplementary material for: Potent and selective inhibition of pathogenic viruses by engineered ubiquitin variants
Source: PLoS Pathog. 2017 May 18;13(5):e1006372. doi: 10.1371/journal.ppat.1006372 (PMC5451084; doi:10.1371/journal.ppat.1006372)
Supplement: S1 Table — (PDF) [file ppat.1006372.s013.pdf]

Table S1. EC_{50} (Ub/UbV) and IC_{50} (UbV) values to cognate viral proteases.

**Ub/UbV**	**EC_{50} (mean)**	**95% confidence intervals**	**Ub-AMC IC_{50} (nM, mean)**	**95% confidence intervals (nM)**	**ISG15-AMC IC_{50} (nM, mean)**	**95% confidence intervals (nM)**
**Ub**	325 **μM**	290 to 363				
**ME.1**	0.7 nM	0.65 to 0.79	3.9	3.52 to 4.27	10.8	6.80 to 17.08
**ME.2**	0.9 nM	0.77 to 0.93	3.0	2.14 to 4.24	16.4	11.94 to 22.57
**ME.3**	4.5 nM	3.31 to 6.00	13.7	10.22 to 18.23	88.9	47.62 to 165.8
**ME.4**	0.2 nM	0.17 to 0.21	0.8	0.75 to 0.91	1.2	1.00 to 1.38
**Ub**	512 **μM**	475 to 552				
**CC.1**	50 nM	42.7 to 58.6	770	11.97 to 49610	Out of range	
**CC.2**	8.9 nM	8.05 to 9.74	10.3	8.76 to 12.12	42.1	32.28 to 52.86
**CC.3**	9.6 nM	9.28 to 9.98	8.8	7.32 to 10.52	102.4	66.26 to 158.1
**CC.4**	0.9 nM	0.85 to 0.93	3.3	2.64 to 4.00	10.9	7.56 to 15.68
**CC.5**	35.6 nM	32.2 to 39.3	390	230.4 to 659.4	Out of range	

Note: In the figures, data are represented as mean ± S.D. of three independent experiments.
