# Supplementary material for: Potent and selective inhibition of pathogenic viruses by engineered ubiquitin variants
Source: PLoS Pathog. 2017 May 18;13(5):e1006372. doi: 10.1371/journal.ppat.1006372 (PMC5451084; doi:10.1371/journal.ppat.1006372)
Supplement: S2 Table — (PDF) [file ppat.1006372.s014.pdf]

Table S2. Binding affinities of viral proteases and UbVs evaluated by Bio-Layer Interferometry (BLI).

Ligands	Analytes	K_D
**CCHFV-OTU**	CC.1	463 ± 44.3 nM
	CC.2	158 ± 11.2 nM
	CC.3	453 ± 17.2 nM
	CC.4	9.0 ± 0.4 nM
	CC.5	10.4 ± 0.7 nM
**MERS-CoV PL^{pro}**	ME.1	19.4 ± 0.7 nM
	ME.2	53.2 ± 2.2 nM
	ME.3	15.6 ± 0.4 nM
	ME.4	35.9 ± 1.6 nM
